# Network-based estimation of therapeutic efficacy and adverse reaction potential for prioritisation of anti-cancer drug combinations

**DOI:** 10.1016/j.csbj.2024.12.003

**Published:** 2024-12-07

**Authors:** Arindam Ghosh, Vittorio Fortino

**Affiliations:** Institute of Biomedicine, University of Eastern Finland, 70210 Kuopio, Finland

**Keywords:** Network medicine, Drug combinations, Computational biology

## Abstract

Drug combinations, although a key therapeutic agent against cancer, are yet to reach their full applicability potential due to the challenges involved in the identification of effective and safe drug pairs. *In vitro* or in vivo screening would have been the optimal approach if combinatorial explosion was not an issue. *In silico* methods, on the other hand, can enable rapid screening of drug pairs to prioritise for experimental validation. Here we present a novel network medicine approach that systematically models the proximity of drug targets to disease-associated genes and adverse effect-associated genes, through the combination of network propagation algorithm and gene set enrichment analysis. The proposed approach is applied in the context of identifying effective drug combinations for cancer treatment starting from a training set of drug combinations curated from DrugComb and DrugBank databases. We observed that effective drug combinations usually enrich disease-related gene sets while adverse drug combinations enrich adverse-effect gene sets. We use this observation to systematically train classifiers distinguishing drug combinations with higher therapeutic effects and no known adverse reaction from combinations with lower therapeutic effects and potential adverse reactions in six cancer types. The approach is tested and validated using drug combinations curated from in vitro screening data and clinical reports. Trained classification models are also used to identify novel potential anti-cancer drug combinations for experimental validation. We believe our framework would be a key addition to the anti-cancer drug combination identification pipeline by enabling rapid yet robust estimation of therapeutic efficacy or adverse reaction potential.

## Graphical Abstract

**Figure:**
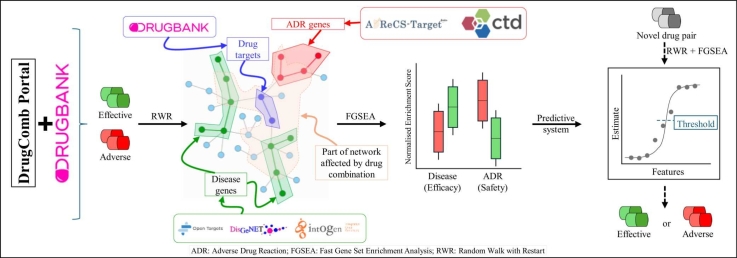

## 1 Introduction

Over the last decades, combination therapy has been crucial in cancer treatments [12], [16]. Their advantage is that they can simultaneously target multiple disease-causing pathways while also reducing individual drug doses [1], [23], [47]. Though the use of lower doses can limit dose-related toxicities, the use of two or more drugs simultaneously can lead to adverse drug-drug interactions (DDI) [3]. Thus, it requires that novel combinations, even involving already approved cancer drugs, are re-screened in vitro and/or in vivo before actual use in the clinic. However, screening all possible combinations is a combinatorial challenge, where the number of experiments required increases exponentially with the number of drugs. Such exhaustive testing is often infeasible [28], [34], leading researchers to prioritize the most promising combinations based on *in silico* tools. Computational tools can screen a large number of drug combinations in a relatively shorter time and prioritize a small number of candidate drug pairs for further pre-clinical and clinical trials [23], [31]. However, a major challenge is developing a robust system that can accurately identify effective and safe drug combinations.

Numerous *in silico* tools, currently available, primarily focus on identifying synergistic or antagonistic drug combinations. They may include monotherapy data, drug-induced gene expression profiles, drug-drug similarity etc. individually or in combination for prediction [10], [14], [17], [21], [44]. While they have been successful in identifying drug pairs that will perform better than the individual drugs alone, existing *in silico* tools do not completely negate the chances of adverse drug-drug interactions (DDI). A separate class of tools is available to predict drug combinations with adverse DDI [13], [18], [4] making this a two-stage problem. This sequential drug combination identification strategy can potentially overlook a critical trade-off between efficacy and safety limiting the selection of good candidates. From a multi-objective optimisation perspective, addressing efficacy first and then safety would imply discarding solutions that might have slightly lower efficacy but much better safety, and vice versa. Furthermore, it is possible that even though a drug combination is synergistic, it may not necessarily lead to sufficient therapeutic efficacy [11], [33]. The lack of efficacy and increased safety concerns have been reported to be the primary causes of low clinical success of drug combinations. At the same time, it is widely recognised that drug combinations even though therapeutically effective might have adverse reactions to some extent [29]. Thus, prioritising drug combinations should strike a balance between therapeutic efficacy and potential toxicities rather than individually focusing on whether drug combinations will have synergistic or adverse interactions. Of late there have been attempts to address the issues within a single task. Especially, advanced machine learning and deep learning-based methods have demonstrated significant success [22], [38], [45]. However, a key limitation has been the amount of data required to train robust models for cancer-type-specific drug combination identification. Another potential disadvantage of such advanced models is their explainability i.e., they often fail to model the relationships of the drug combination targets to the known disease and adverse events associated genes and then use the information to establish exact rules that clearly distinguish effective and adverse drug combinations.

Network-based data mining algorithms using protein-protein interaction (PPI) networks that cover known drug targets, genes/proteins dysregulated by the disease, and those associated with adverse drug reactions could provide an overall solution to the problem [31]. Network-based approaches have been successfully used to develop *in silico* strategies for the identification of effective and safe drug combinations. For example, [43] proposed a novel score to quantify the efficacy and safety of drugs or their combinations based on the expression of disease-related genes and essential genes within the sub-networks affected by drugs or their combinations. On a limited number of Type 2 Diabetes mellitus drug combinations, they showed that an effective drug combination has a higher score than its constituent drugs. While this approach effectively integrates transcriptomic data with known biomolecular interactions, it restricts the identification of drug combinations to those for which the gene expression of the individual drugs is available. To alleviate this bottleneck, [5] utilised the drug combination targets to identify the afflicted pathways and devised a synergy score that considers the topological associations between them in a pathway-pathway interaction network. They concluded that synergistic drug combinations are formed by drugs that either act on the same pathway through different targets or regulate a small number of highly connected pathways. A key limitation here is the reliance on known pathways and their interconnectedness for evaluating synergy scores. In a similar drug target-based approach, instead of the connections between the pathways, [6] used shortest paths between the drug combination targets and the disease-related genes to calculate a separation distance that can effectively identify disease-specific clinically efficacious drug combinations. According to their findings, for drug combinations to be effective, not only should the drugs have distinct sets of genes/proteins as targets, but they should also target the same disease module. This approach potentially limits itself by using the shortest path to gauge proximity between two gene sets, neglecting significant indirect interactions and broader network context [36]. Moreover, a single metric used to measure the distance between known drug targets and disease genes is accounted for distinguishing both safe and unsafe drug combinations. While this disease-centric approach might be sufficient to account for therapeutic efficacy it undermines the role of genes/proteins that are linked to adverse effects. This limitation suggests the need to use both disease-related genes/pathways and adverse-effect-related genes/pathways to comprehensively evaluate the efficacy and safety of drug combinations, ensuring that proximity to therapeutic targets does not overshadow potential risks associated with adverse reactions.

This study presents a novel computational approach that integrates network propagation algorithms, like Random Walk with Restart (RWR) [36], with gene set enrichment analysis to quantify the potential therapeutic efficacy and adverse-effect-related safety of drug combinations. This integration goes beyond simply considering the shortest paths between drug targets and disease/adverse-effect genes/pathways. RWR leverages the network structure to comprehensively explore and rank intermediate genes (potentially involved in the drug's mechanism) connecting drug targets to disease or adverse effect genes. It thus provides a more robust ranking of network genes compared to traditional shortest-path algorithms. Furthermore, the incorporation of gene set enrichment analysis reveals if the ranked genes are enriched within disease gene sets, adverse effect gene sets, or potentially both. Subsequently, the enrichment scores could be used as predictors to identify drug combinations that are effective against the disease while posing a lower risk of adverse effects. We systematically evaluate our *in silico* approach by leveraging existing data on cancer cell lines [46] and reported adverse DDI from DrugBank [40] to classify drug combinations as "effective" (higher therapeutic efficacy with no adverse reaction from DDI) or "adverse" (lower therapeutic efficacy with adverse reaction from DDI). Initial assessment through statistical method revealed that effective drug combinations had a higher enrichment of disease-related gene sets, whereas adverse drug combinations had a higher enrichment of adverse-effect-related gene sets. This observation was leveraged to develop predictive systems for identifying and prioritizing effective and safe drug combinations across six cancer types followed by validation using known labelled drug combinations. Additionally, these predictive systems were used to identify novel anti-cancer drug combinations suitable for further experimental validation.

## 2 Materials and methods

### 2.1 Summary of the proposed computational workflow

**Fig. 1:**
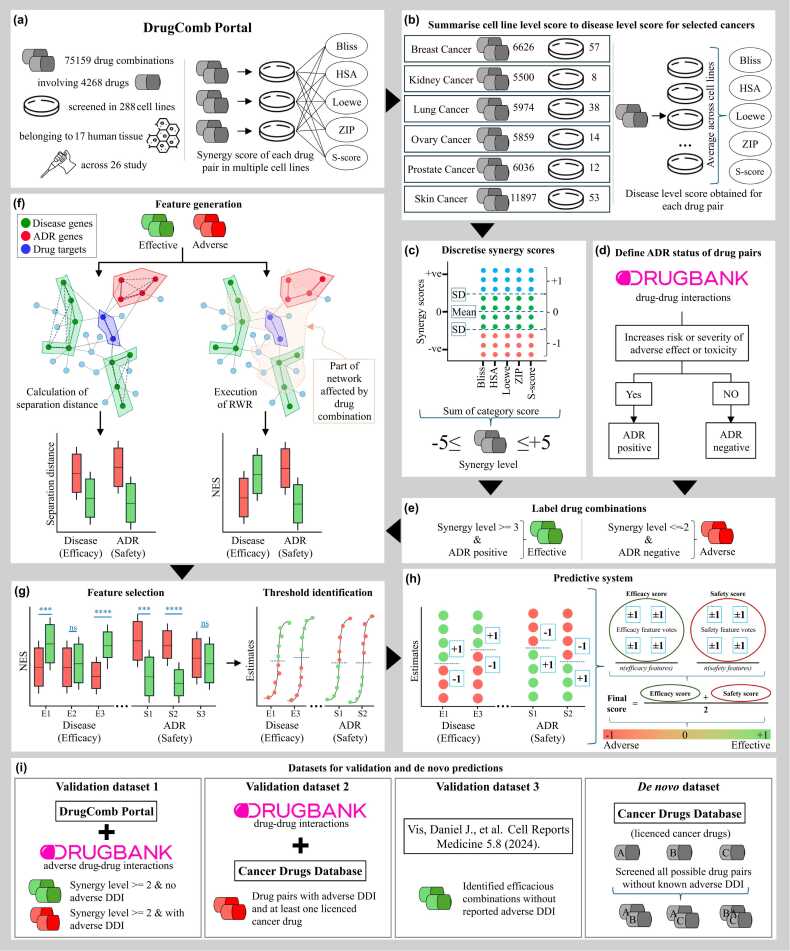
Overview of the network-based pipeline for identification of effective and adverse drug combinations. The panels (a) – (h) illustrate the steps used for training the predictive system starting from the raw data. Two primary data sources are used for building the training data – drug combination screening data from the FIMM DrugComb portal (in (a)) and DDI reports from DrugBank (in (d)). The panel (a) summarises the type of data available in the portal while panels (b) and (c) summarise the steps used to convert the cell line level synergy scores first to a disease level synergy score and finally to a discretised synergy level for the drug combinations. The panel (d) shows the criteria for defining the ADR status of the drug combinations. The two data are merged in panel (e), to curate the training dataset. This training dataset is used to compare our RWR-FGSEA-based approach with a direct network proximity-based approach as shown in panel (f). It also hints at the kind of pattern observed in the comparison result. The panels (g) describe how the features that show statistically significant differences in NES between the effective and adverse drug combinations selected followed by the identification of threshold NES that best differentiates the two classes of drug combinations. The thresholds identified are then used in panel (h) to perform an initial classification of the drug combinations based on each feature followed by combining the votes to obtain a final score that is used to assign the predicted class of the drug combinations. The panel (i) shows sources of the validation and *de novo* datasets. It also describes how the combinations are labelled in the validation data and how the combinations are generated for the novel drug screening. For prediction on the validation data or *de novo* data, the pipeline requires only executing the RWR-FGSEA step (part of panel (f)) followed by using the thresholds to perform the voting based on each feature and calculating the final score (panel (h)).

The framework uses drug target information to derive a surrogate estimate of the potential of the drug combinations to induce therapeutic efficacy or cause adverse effects. RWR, a network propagation algorithm, is first used to identify the key genes/proteins within a PPI network affected by the drug combinations. Subsequently, fast gene set enrichment analysis (FGSEA) is applied to verify whether these genes/proteins are enriched in disease-related gene sets, adverse-effect-related gene sets, or both. The normalised enrichment scores (NES) from FGSEA, alternately referred to as efficacy (for enrichment against disease-related gene sets) or safety (for enrichment against adverse-effect-related gene sets) estimates, are used to compare effective and adverse drug combinations. We define effective drug combinations as those that have potentially higher efficacy with no reported adverse DDI while adverse drug combinations as those that have lower therapeutic efficacy with known adverse DDI. The ability of these estimates to identify potential new drug combinations is assessed using several datasets of labelled drug pairs for treating different cancer types. These datasets are derived from drug combination screening data in cancer cell lines assessing synergism or antagonism [37], [46], and clinical reports of adverse reaction-inducing DDI from the DrugBank [40]. We compare our approach to an established framework of using direct proximity-based separation distance for prioritising effective drug combinations. Finally, we use our approach to develop predictive systems and identify novel anti-cancer drug combinations using licensed anti-cancer drugs. Fig. 1 graphically illustrates this proposed network medicine-based computational framework to rank and identify novel drug combinations for cancer treatments.

### 2.2 Labelling anti-cancer drug combinations using drug combination screening data and clinical reports of drug-drug interactions

**Fig. 2:**
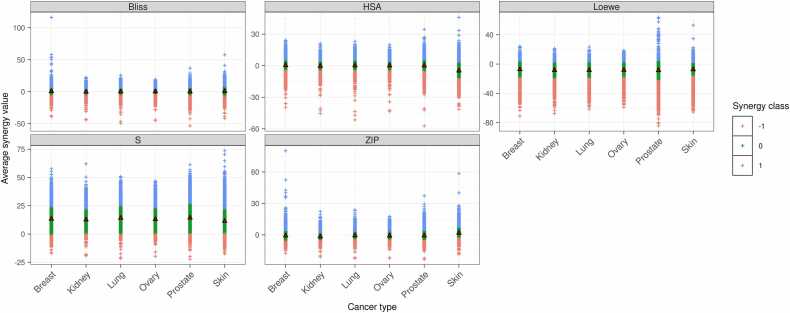
Classification of drug combinations from the DrugComb portal into synergistic (+1), additive (0) and antagonistic (−1) types. For the drug combinations in the portal, the average synergy score across multiple cell lines of the same cancer type was computed followed by calculating the expected mean synergy score for each cancer type. This expected mean synergy score and the corresponding standard deviation within each score type was used as the basis for classification. The drug combinations with synergy scores above 0 and greater than the mean increased by one standard deviation were assigned as synergistic. Alternately, the drug combinations with synergy scores below 0 and less than the mean decreased by one standard deviation were assigned as antagonistic. The drug combinations that did not fall within this criterion were assigned as additive.

In the absence of a gold-standard dataset, effective and adverse drug combinations were defined using in vitro drug combination screening data on cancer cell lines and reported adverse reaction-inducing DDI. An overall synergistic score was systematically computed from five different synergy scores – namely Bliss, highest single agent (HSA), Loewe, zero interaction potency (ZIP) and S-Score – of drug combinations in cancer cell lines. These scores were obtained from the FIMM DrugComb portal [46] which collects data from several independent drug combination screening studies and provides standardised and harmonised synergy scores for each drug pair in each cell line. The cell lines were mapped to their respective diseases using the NCI thesaurus and those for cancer were retained. Then, for each scoring model, the average synergy score for each drug pair across multiple cell lines of the same cancer type was computed followed by calculating the expected mean synergy score for each cancer type. This expected mean synergy score was used as a baseline of synergy to classify drug combinations as synergistic, additive or antagonistic represented by + 1, 0 and −1 respectively (Fig. 2). Precisely, drug pairs with synergy scores above 0 and greater than the mean plus one standard deviation were assigned a score of + 1. Conversely, those with synergy scores below 0 and lower than the mean minus one standard deviation were assigned a score of −1. The drug pairs that do not fall within this criterion were assigned a score of 0. This new discretised score from five different scoring models was summed to obtain a synergy level for the drug pairs. The synergy level, which can range between −5 to + 5, reflects the confidence of the drug combination to be antagonistic or synergistic respectively. Parallelly, all known reports of interactions between drug pairs were retrieved from DrugBank (version 5.1.10) [40]. These interactions, recorded as statements, could be either beneficial or adverse. The drug combinations that were reported to have an increased risk or severity of adverse effects, liver damage or toxicities were labelled as “adr_positive” or else “adr_negative”. The R function ‘grep’ was used to perform this check with the pattern “risk or severity of.+toxicity can be increased”, “risk or severity of liver damage can be increased”, “risk or severity of adverse effects can be increased”, and “increase the.+toxic activities”. These search patterns were designed to retrieve all the potentially high-risk adverse effects relevant to drug combination treatment. Finally, the synergy level and the ADR status were merged to categorize the drug combinations as effective or adverse. The drug pairs with synergy levels greater than or equal to + 3 and no reported adverse DDI (i.e., adr_negative) were classified as effective while those with synergy levels less than or equal to −2 and with reported adverse DDI (i.e., adr_positive) were classified as adverse. Table 1 reports the number of effective and adverse drug combinations selected for each cancer type. These selected drug combinations were used to assess the goodness of the network distance-based classification systems for prioritising drug combinations.

**Table 1:** Number of drug combinations in the training data. The table also provides the distribution of the drug combination counts across different synergy levels which formed the basis for classifying the drug combinations as effective and adverse. Also listed are the different drug-drug interaction types for the adverse drug combinations.

Cancer	Effective	Adverse	Synergy levels	DDI types for adverse
Breast Cancer	72	52	5 = 6; 4 = 27; 3 = 39; −2 = 28; −3 = 11; −4 = 7; −5 = 6	risk or severity of adverse effects can be increased = 52
Kidney Cancer	69	44	5 = 7; 4 = 28; 3 = 34; −2 = 26; −3 = 12; −4 = 3; −5 = 3	risk or severity of adverse effects can be increased = 44
Lung Cancer	92	45	5 = 16; 4 = 35; 3 = 41; −2 = 22; −3 = 13; −4 = 7; −5 = 3	risk or severity of adverse effects can be increased = 44, risk or severity of cardiotoxicity can be increased = 1
Ovary Cancer	93	43	5 = 16; 4 = 30; 3 = 47; −2 = 22; −3 = 8; −4 = 10; −5 = 3	risk or severity of adverse effects can be increased = 41; risk or severity of cardiotoxicity can be increased = 2
Prostate Cancer	73	59	5 = 11; 4 = 26; 3 = 36; −2 = 29; −3 = 22; −4 = 6; −5 = 2	risk or severity of adverse effects can be increased = 59
Skin Cancer	82	41	5 = 17; 4 = 32; 3 = 33; −2 = 29; −3 = 7; −4 = 4; −5 = 1	risk or severity of adverse effects can be increased = 41

### 2.3 Retrieval of drug targets

The targets of the drugs involved in forming the combinations were retrieved from the DrugBank database (version 5.1.10) [40]. DrugBank reports both intended and unintended targets of the drugs. More specifically, DrugBank identifies a molecule, including both proteins and nucleic acids from humans or other organisms, as a target if it is involved in the transport, delivery or activation of the drug [41]. The drug targets are primarily identified by a literature search in PubMed [42]. They are additionally confirmed based on information from other databases like FDA labels, RxList, PharmGKB and Therapeutic Target Database (TTD) which in turn reports drug-targets associations based on manufacturer-submitted information, clinical reports, pharmacogenomics etc. In our study, only the human protein targets were retained for analysis.

### 2.4 Preparation of protein-protein interaction network

A PPI network from the STRING (version 12.0) database [35] was used to study the relationship between drug targets, disease genes and safety-related genes. In STRING, each PPI is annotated with seven ‘scores’, each determined based on different types of information from three different contexts – genomic context, functional genomic experiments or direct lab assays and prior knowledge. In our study, all possible interactions between human proteins in the database were retrieved and filtered to retain those with a combined score greater than 400 (equivalent to 0.4 in scaled combined score).

### 2.5 Gene sets for measuring proximity between drug targets and disease or safety-related genes

The disease-related gene sets were retrieved from different databases like DisGeNET (release May 2020) [27], Intogen (release 31–05-2023) [24], Open Targets (v23.09) [25] and PharmGKB [39]. In addition, the GEO signatures from the Enrichr [20] database and the top 50 genes prioritized by TheTA [9], a tool to identify novel disease-associated genes, for cancer were also compiled. Only the curated gene-disease associations from DisGeNET and direct associations based on human genetics data, literature, and RNA expression profiles from Open Targets were included. Since these databases do not always include gene-disease associations for the primary cancer, the genes associated with the cancer sub-types were included. The specific gene sets were selected from the databases such as to include the tissue name e.g. “breast” and terms “cancer” or “carcinoma” or “sarcoma” in their names while excluding gene sets containing the terms “hereditary” or “familial” or “susceptibility” or “predisposition”. Notably, multiple different sources were included to avoid any specific biases due to the gene-disease association curation methods. Moreover, some of these data sources focus on specific molecular information: for example, DisGeNET focuses on genetic information, while GEO signatures are based on transcriptomic data. This inclusion thus enables the identification of key molecular aspects relevant to distinguishing effective from adverse drug combinations. Similarly, the adverse-effect-related gene sets were retrieved from databases like ADReCS-Target [15], Comparative Toxicogenomics Database (CTD) (release November 2023) [8], and DisGeNET (release May 2020). These gene sets were identified based on six adverse reaction terms – namely hepatotoxicity, immune-related reaction, cardiotoxicity, neurotoxicity, haematological toxicity, and carcinogenicity – reported in the literature to be frequently associated with post-marketing drug withdrawal [2]. Additional gene sets containing terms like “adverse reaction”, “toxicity” and/or “drug” in their names were also manually extracted and compiled as safety-related gene sets. Supplementary File 1 lists the sizes of each gene set, highlighting that some may have many associated genes, while others are more specific. This helps assess if gene set size impacts network-based estimation of efficacy or safety.

### 2.6 Estimation of efficacy and safety of drug combinations

The framework uses a combination of RWR [36] and FGSEA [19], an approach we previously used for addressing different computational challenges [30], [32], to quantify efficacy and safety. In theory, RWR explores the global network structure and calculates the proximity of a set of given ‘seed’ nodes to all nodes in the network. These proximities, also referred to as visiting probabilities (higher is closer), can be used to rank the nodes for subsequent use as input for FGSEA to evaluate their significance in the context of predefined gene sets. In our study, drug targets of each drug combination were used as seed nodes to run the RWR algorithm as implemented in the R package dNet (version 1.1.7) with a restart probability of 0.5. A threshold was then selected using the elbow method from the resultant probabilities and genes/proteins exceeding the threshold used to perform FGSEA using the fgsea (version 1.24.0) R package. To explain in more detail, the non-seed node probabilities were plotted in descending order, followed by identifying the reference line connecting the plot's largest and smallest probability points. The perpendicular distances of all the probability points to the identified reference line were evaluated and the probability corresponding to the highest perpendicular distance was considered as the threshold. The FGSEA was executed against a combined set of disease and adverse effect-related gene sets with minSize = 5, maxSize = 500 and scoreType = “pos” as the parameters. Here, the use of scoreType = “pos” ensures the identification of overrepresented gene sets and prioritises the genes with high visiting probabilities. The obtained NES was considered the efficacy and safety estimate of the drug combinations.

### 2.7 Calculation of direct proximity

In our study, the estimates from the RWR-FGSEA approach were compared to a baseline shortest path-based approach to verify if the former would outperform the latter in distinguishing effective from adverse drug combinations. While different network-based proximity measures exist, the method proposed by [6] called separation distance was chosen as it has been shown to outperform similar methods.

### 2.8 A classification system for anti-cancer drug combinations using network medicine and gene sets linked to disease physiology and adverse drug reactions

This section describes the classification system for distinguishing effective from adverse anti-cancer drug combinations. Both statistical and machine learning-based analyses are employed to explore if the efficacy and safety estimates derived from the union of known targets of two drugs are potentially good predictors of effective and adverse drug combinations.

*Assessing the difference between efficacy and safety estimates:* The Wilcoxon test was used to determine if estimates (or NES) from individual gene sets are sufficient to classify the drug combinations. Through a one-sided test, we specifically checked if the NES for effective drug combinations is greater in disease-related features and if the NES for adverse drug combinations is greater in adverse-effect features. This is based on our assumption that effective drug combinations should be closer to or have more influence on disease-related gene sets than adverse-effect-related gene sets and vice versa. The gene sets showing statistical significance (at p < = 0.05) were selected for the subsequent machine learning analysis. The Wilcoxon test was also used to check the informativeness of the separation distances for distinguishing the drug combinations. However, in this case, a two-sided test was used as ideally the targets of effective drug combinations should have a lower separation distance to the gene sets irrespective of the feature type.

*Training a robust and highly interpretable classification system for distinguishing effective from adverse drug pairs:* A hierarchical classification system was implemented for distinguishing effective from adverse drug combinations. In the first stage, for each cancer type, a logistic regression classifier is trained using the estimates of each selected feature assigning 1 to the positive class i.e., effective drug combinations and 0 to the other. This training data is then used to generate predictions using the resultant logistic regression model. The sensitivities and specificities for the complete probability range generated from the model are calculated and the one corresponding to the highest balanced accuracy is identified. The inverse logistic regression function is ultimately used to solve for the NES value that would serve as the threshold for distinguishing the drug combinations. The identified thresholds are used to assign an initial classification of the drug combinations within each feature into effective and adverse represented by the votes + 1 and −1 respectively. However, the different feature types use a slightly different voting scheme. Drug combinations receive a + 1 vote for disease-related features if their NES meets or exceeds the threshold, otherwise −1. For adverse-effect-related features, the voting is reversed. In the second stage, efficacy and safety scores are calculated as the average of votes within the disease-related and adverse-effect-related features respectively. A final score is then calculated by averaging the efficacy and safety scores. The final score determines the final classification of the drug combinations. A drug combination is labelled effective if the score is positive else it is labelled adverse. The proposed hierarchical classification system offers several advantages. The clear cut-off based on the gene set enrichment makes the system highly interpretable. Averaging predictions from multiple classifications, enhances robustness, reducing the impact of individual errors for more reliable results. Its balanced two-step integration of efficacy and safety assessments ensures comprehensive decision-making while also ensuring the influence of each feature group is normalised to mitigate biases due to an unbalanced number of features (e.g., more disease-related gene sets), leading to fairer and more accurate predictions. The final predictive systems developed in our study utilise the NESs from the complete set of effective and adverse drug combinations within each cancer type. However, we implement ten iterations of a repeated three-fold cross-validation framework to assess system variability. At each iteration, the dataset was split into three folds containing equal proportions of effective and adverse drug combinations. Two of these folds were used to train the predictive system involving the aforementioned steps of fitting a logistic regression model and identifying threshold NES for each feature. The third left-out fold was used as a test set to measure the accuracy of the predictive system. The thresholds determined from the training fold were used as the basis for initial feature-wise voting on the test set data followed by calculating the efficacy, safety and final score. The predictive models were evaluated using three metrics: balanced accuracy, sensitivity and specificity.

### 2.9 Validation against sets of drug pairs not used for the training datasets

For prediction on novel datasets, the system directly uses the identified efficacy and safety estimate thresholds to execute the voting scheme on each feature followed by calculating the efficacy, safety and final scores to assign classes to the drug combinations. Three datasets consisting of pairs of small molecular drugs with known targets in the PPI network were curated to assess the generalisability of the developed predictive systems. The first dataset (Validation Data 1) is a subset of the FIMM DrugComb portal drug combination data that did not qualify as the training set. The drug pairs with synergy levels greater than or equal to + 2 and no reported adverse DDI (i.e., adr_negative) were labelled effective while those with synergy levels greater than or equal to + 2 and reported adverse DDI (i.e., adr_negative) were labelled adverse for this dataset. Although less potent, these drug combinations may still be clinically significant. The second dataset (Validation Data 2) consisted of only adverse drug combinations to verify the model’s ability to correctly identify the negative class. For this dataset, the drug pairs involving at least one known licensed anti-cancer drug and having reports of an increased risk of toxicity or adverse effects were extracted from DrugBank. The list of cancer-specific licensed anti-cancer drugs was retrieved from the Cancer Drug Database [26]. The third validation data (Validation Data 3) was derived from a recently published in vitro study that screened 51 drug combinations in cancer cell lines from 21 tissue types to identify clinically beneficial combination treatments. The reported efficacious drug combinations for five cancer types and the respective cell lines that overlapped with our study were considered to build the dataset. To extrapolate cell line-level efficacy to the tissue level, a drug combination was deemed effective for a tissue if it exhibited efficacy in one or more of its constituent cell lines. All the drug combinations thus derived were labelled as effective except for combinations that had previous reports of an increased risk of toxicity or adverse effects in DrugBank. In those cases, the drug combinations were labelled as adverse. The validation dataset, however, ended up with only effective drug combinations after applying the filters described in this section. This dataset was included to assess the models' ability to identify true positives correctly. In all three datasets, the drug combinations were filtered to remove those that were used in training the predictive models. It must be noted that due to this filtration, the effective drug combinations in validation data 1 only consist of those with synergy level + 2. Furthermore, for the first two validation datasets, only drug combinations whose paired chemical subgroup (defined by ATC code level 4) matched with the paired chemical subgroup of the drug combinations in the training dataset were retained (Appendix A, Appendix A). For instance, if the training data only includes combinations of 'taxanes' and 'sulfonamides,' the validation data was restricted to combinations of the same drug classes. This filter was however not applied for the third dataset as it reduced the number of drug combinations to zero. The number of drug combinations for each category in these datasets is summarised in Table 2.

**Table 2:** The number of drug combinations in the validation and *de novo* data sets.

Dataset	Breast Cancer		Kidney Cancer		Lung Cancer		Ovary Cancer		Prostate Cancer		Skin Cancer	
	Adv	Eff	Adv	Eff	Adv	Eff	Adv	Eff	Adv	Eff	Adv	Eff
Validation Data 1	9	5	6	5	12	11	8	11	19	11	16	18
Validation Data 2	108	-	37	-	84	-	103	-	68	-	34	-
Validation Data 3	-	18	-	16	-	17	-	11	-	-	-	11
De Novo	731		792		960		1132		770		921	

### 2.10 *De novo* prediction dataset

A dataset of all possible combinations of small molecular licensed anti-cancer drugs approved by the Food and Drug Administration (FDA) and European Medicines Agency (EMA) was created for screening novel drug combinations. The individual drugs used for curating this dataset were retrieved from the Cancer Drug Database. Similar to the validation datasets, these combinations were also filtered to retain only those combinations whose paired chemical subgroup (defined by ATC code level 4) matched with the drug combinations’ paired chemical subgroup in the training dataset (Appendix A, Appendix A). However, the exact combinations that have been used for training the predictive model were removed. The dataset was also filtered to remove drug combinations that have already been reported to have an increased risk of toxicities or adverse effects and those that have already entered clinical trials of the specific cancer type.

## 3 Results

### 3.1 Characterization of a drug combinations search space for cancer research

We utilised the drug combinations collated in the FIMM DrugComb portal as the starting point of our analysis. The drug combinations were retrieved and processed as described in the materials and methods sections to identify a robust dataset of effective and adverse drug pairs for six cancer types, including breast, kidney, lung, ovarian, prostate, and skin cancers. These six cancers were selected as they formed the bulk of data in the portal. Table 1 shows the number of drug combinations finally shortlisted for our analysis. It must be mentioned here that the relatively small number of drug combinations can be attributed to the strict filtering we applied for identifying the drug combinations. Among all the drug combinations screened, most had synergy levels between −1 and + 1 implying that they may be classified as synergistic based on one synergy model but antagonistic or neutral based on another (Supplementary Figure 1). Further, only 25% of the drug combinations within each cancer type were reported to have severe adverse drug-drug interactions. Hence, we selected the drug combinations at the extreme ends of the spectrums when considering the synergy level and ADR status axes. This way we prioritise drug combinations that have been experimentally shown to be effective, while also prioritising those with known relevant adverse drug-drug interactions. Interestingly, we observed that most drug combinations in the final dataset were specific for a particular cancer type (Supplementary Figure 2). They were mostly formed by combining kinase inhibitors with taxanes (Appendix A, Appendix A). Also, most individual drugs that participated in forming the combinations in the final dataset were distributed among both effective drug combinations and adverse drug combinations (Supplementary Figure 3). Notably, adverse drug combinations in the final dataset were mostly associated with increased risk or severity of adverse effects without the mention of the specific adverse effect that the drug combination might cause.

### 3.2 Estimating the efficacy and safety of anti-cancer drug combinations using RWR-FGSEA as a network proximity measure

**Fig. 3:**
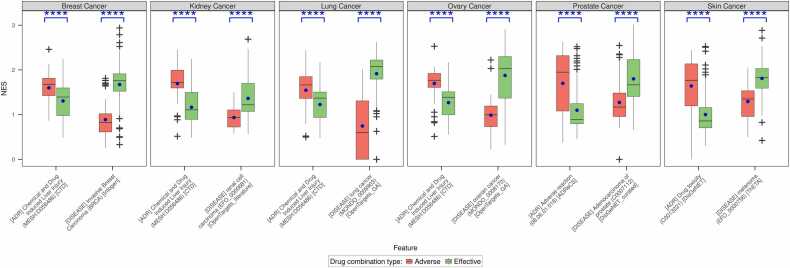
Distribution of the efficacy and safety estimates of effective and adverse drug combinations. The efficacy and safety estimates of the two groups of drug combinations (adverse drug combinations: red vs. effective drug combinations: green) obtained from the RWR-FGSEA step were compared using Wilcoxon’s test to check if they are informative in distinguishing effective from adverse drug combinations. The features that were statistically significant in the statistical test were used to develop the predictive system. Shown here are one efficacy feature and one safety feature from each cancer type that passed the statistical test and had the least p-value within each feature type. The blue dot within each box shows the mean value. The asterisks on top of each pair of boxes indicate the level of significance (***: p < = 0.001; ****: p < = 0.0001).

The RWR-FGSEA pipeline was executed on the effective and adverse drug combinations shortlisted in the previous section. Based on initial analysis, we observed that in most cases the mean NES for effective drug combinations was higher for the efficacy-related gene sets while the mean NES for adverse drug combinations was higher for the safety-related gene sets (Supplementary Figure 4). This was also statistically confirmed by Wilcoxon’s test. In line with our expectations, we observed that there was at least one efficacy-related gene set, and one safety-related gene set in each cancer type that showed statistically significant differences (at p < = 0.05) in the distribution of the NES (Fig. 3). However, there were a few exceptions to the pattern. Instead of the adverse drug combinations, the effective drug combinations showed higher enrichment for the cardiotoxicity and the neurotoxicity gene sets. This result might be due to their similarity to efficacy or disease-related gene sets. Notably, the effective drug combinations had no reported adverse drug-drug interactions which necessarily does not infer that there would be no adverse reactions due to the long-term usage of the drug combinations. Similarly, for cases where the adverse drug combinations show higher enrichment of efficacy gene sets compared to effective drug combinations, it is possible that the effective drug combinations included in the training set do not explicitly target close to the genes within that gene set. It must be noted that the gene sets were curated to accommodate different molecular aspects of the disease and might include subtypes of primary cancer. Furthermore, we observed that the safety-related gene sets Adverse reaction (08.06.01.018) [ADReCS], Chemical and Drug Induced Liver Injury (MESH:D056486) [CTD] and Drug toxicity (C0013221) [DisGeNET] were common for all the cancer types (Appendix A, Appendix A). Apart from these, the gene set for Chemically-Induced Liver Toxicity (C4279912) [DisGeNET] also showed statistically significant differences in the distribution of the estimates in four cancers. The frequent occurrence of the non-specific terms is a reflection of the adverse drug combinations included in our study i.e., they were not associated with any specific adverse reactions. This highlights the importance of the inclusion of adverse drug combinations with specific toxicities for robust identification of effective and adverse drug combinations. In the case of the efficacy-related features, only the gene sets built using TheTA showed significance in all six cancers. The gene sets sourced from Intogen, OpenTargets (genetic association and somatic mutation) and DisGeNet (curated) were significant in five of the six cancers in the study. It must also be noted that not all the sources were represented in all cancers. For example, there were no gene sets from Intogen and OpenTargets (somatic mutation) for ovary and kidney cancer respectively. Despite this, it can be observed that disease-related gene sets based on genetic associations are prioritised for the separation of effective from adverse drug combinations.

### 3.3 Comparison of efficacy and safety estimates with a direct network-based proximity method

**Fig. 4:**
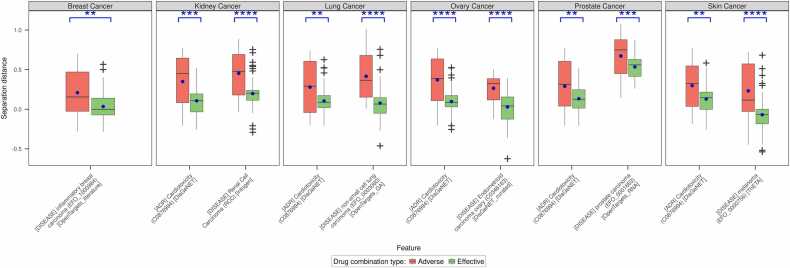
Distribution of the separation distances (proximities) between the targets of effective drug combinations (reported as green boxplots) and adverse drug combinations (reported as red boxplot) and the efficacy and safety gene sets (features). Wilcoxon’s test was also used to assess the informativeness of the separation distance of the drug combination targets to the disease-related and adverse-effect-related genes in distinguishing effective from adverse drug combinations. Shown here are one efficacy feature and one safety feature from each cancer type that passed the statistical test and had the least p-value within each feature type. If a feature of any one type is not represented, it infers that there were no significant features. The asterisks on top of each pair of boxes indicate the level of significance (***: p < = 0.001; ****: p < = 0.0001).

We observed that the separation distances of the effective drug combination targets to either efficacy or safety-related gene sets were consistently shorter compared to the distance from adverse drug combination targets (Supplementary Figure 5). This was in line with previous findings that targets of effective drug combinations must overlap with the disease-related genes. Wilcoxon test showed that except for breast cancer, all others had at least one efficacy and one safety feature for which the distribution of the separation distances was significantly different (at p < = 0.05) (Fig. 4). The cardiotoxicity gene set from both DisGeNET (C0876994) and CTD (MESH:D066126) were common in all the five cancers that showed this pattern (Appendix A, Appendix A). Interestingly, the gene sets for Adverse reaction (08.06.01.018) [ADReCS], Chemical and Drug Induced Liver Injury (MESH:D056486) [CTD] and Drug toxicity (C0013221) [DisGeNET] that had a significant difference in NES in all cancers, do not repeat the pattern with proximity. The gene sets Chemical and Drug Induced Liver Injury (MESH:D056486) [CTD] and Chemically-Induced Liver Toxicity (C4279912) [DisGeNET] were observed to have significantly different proximity in kidney and ovary cancers only. Concerning the efficacy gene sets, TheTA again emerged to be common among all cancers while the gene sets sourced from Intogen, OpenTargets (genetic association and RNA) and DisGeNet (curated) occurred as significant in five of the six cancers. Based on these observations, it can be hypothesized although the proximity to efficacy-related gene sets might be sufficient it might be more appropriate to quantify how effective a drug combination would be – lower proximity inferring higher effectiveness. Unlike the direct network-based proximity method, RWR-FGSEA suggests that drug combinations must satisfy two criteria simultaneously to be classified as effective or adverse. Specifically, the effective drug combinations must have a higher efficacy estimate but a lower safety estimate, while the adverse drug combinations must have a higher safety estimate and a lower efficacy estimate. Ideally, the gap between these two estimates could dictate how effective a drug combination would be without causing any side effects.

### 3.4 A predictive system for distinguishing effective from adverse cancer drug combinations

We leveraged the observation about differentially enriched efficacy and safety gene sets between the effective and adverse drug combinations to develop a threshold-based system to predict labels for novel cancer drug combinations. Effectively, these thresholds (Supplementary Figure 6) provide a baseline to understand how high the NES of a drug pair against a particular gene set should be for it to be classified as effective or adverse. We observed an average threshold NES of 1.223 across all cancer and feature types. Practically, for a novel drug combination to be effective, its NES against efficacy gene sets should be higher than 1.223 while its NES against safety gene sets should be lower than 1.223. If the NES exceeds the threshold values against both efficacy and safety gene sets, it will inform that the drug combination although highly effective also has a high chance of causing adverse reactions. Ideally, it would be desirable to identify drug pairs that have higher NES against efficacy gene sets and lower NES against safety gene sets.

**Fig. 5:**
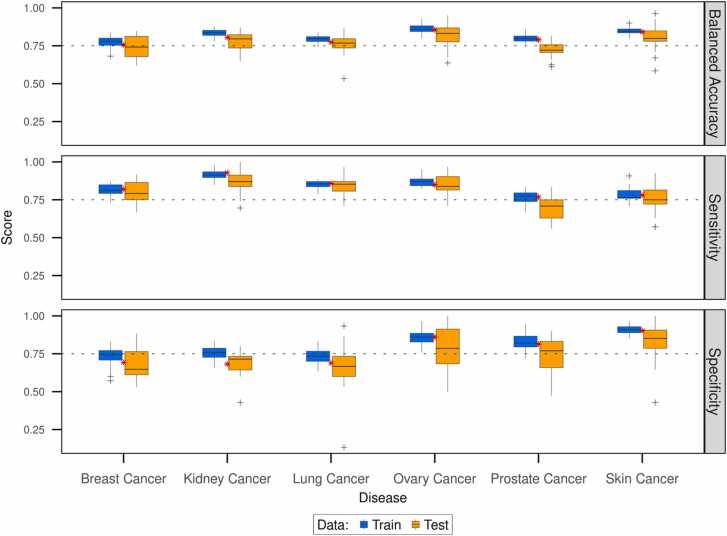
Accuracy of the predictive system. The efficacy and safety estimates obtained from the RWR-FGSEA approach were used to develop predictive systems to distinguish effective from adverse drug combinations in each cancer type. The box plots here show the accuracy scores of the systems on training (blue boxplots) and test (orange boxplots) partitions of the data during the ten iterations of three-fold cross-validation. The red asterisk shows the final accuracy score of the predictive system trained on the complete dataset.

From the variability assessment of the predictive systems using a cross-validation framework, we observed that the threshold selected based on the complete data was either equal to or close to the median threshold identified over the performed ten iterations (Supplementary Figure 7). It must be mentioned here that predictive systems presented in the study use thresholds based on the complete dataset (Supplementary Figure 6). Further, we observed that the median balanced accuracy for both the training and test data was above 0.75 for most cancer types (Fig. 5). A deviation was observed in the case of balanced accuracy on the test data for breast cancer and prostate cancer where it remained slightly below the 0.75 mark. Additionally, the predictive systems for breast and lung cancer had a median specificity (true negative rate) below 0.75 but were compensated by the higher sensitivity (true positive rate) during cross-validation. On the final predictive system based on the complete dataset though, we observed that the predictive systems for all cancer types achieved a minimum balanced accuracy of 0.75. Consistent with the variability check using the cross-validation framework, we observed that even in the final predictive system, the breast, kidney and lung cancer systems had a lower specificity but were compensated by higher sensitivity.

### 3.5 Assessment of the predictive system on external datasets

**Fig. 6:**
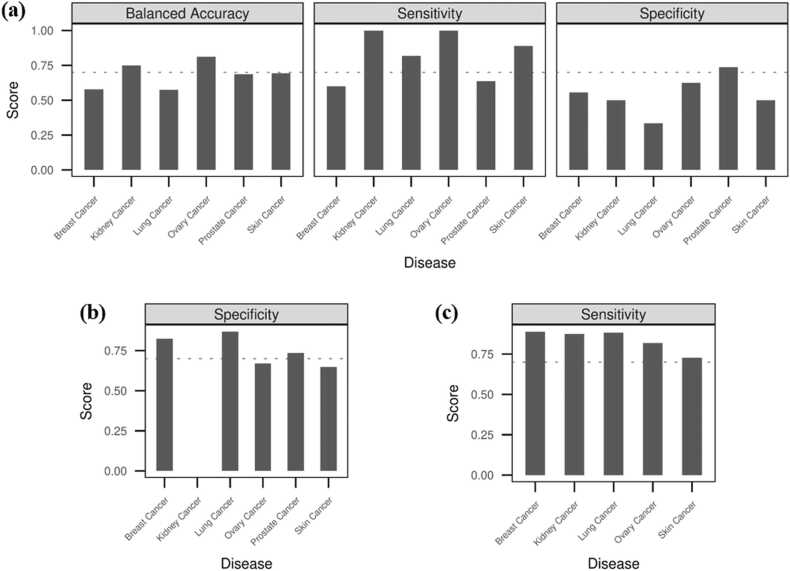
Accuracy of the predictive systems on the external validation datasets. For the validation dataset 1 (fig. a), sensitivity, specificity and balanced accuracy were measured. For the validation datasets 2 and 3 (fig. b and c), only specificity and sensitivity were measured respectively as they included only negative and positive samples respectively. The absence of a bar indicates a zero score.

The proposed predictive system for identifying and ranking cancer drug combinations was assessed against sets of untested drug combinations, labelled either effective or adverse. The assessment was primarily carried out within the framework of possible chemical subgroup pairing in the training data. When evaluated against the validation data 1, the predictive system showed a moderate performance achieving a mean balanced accuracy of 0.68 (Fig. 6a). Finer inspection revealed that the predictive system could correctly predict the true positives (mean sensitivity = 0.82) but had difficulty predicting the negative class correctly (mean specificity = 0.54). This was interesting because the predictive system seems to identify the drug combinations as effective in most cases. This could be considered in line with the fact that all drug combinations included in this dataset had synergy level + 2 or more meaning that they were found to be synergistic by at least two synergy scoring models. Yet the inaccuracy in detecting the negative class could be of concern as predicting adverse drug combinations as effective could have detrimental consequences. To better understand if the predictive system consistently fails in detecting negative class, the system was assessed using only negative labels i.e., only adverse drug combinations (Validation Data 2). It was observed that the breast and lung cancer systems correctly identified the drug combinations as adverse in more than 80% of the cases (Fig. 6b). The prostate cancer system was not far behind and showed correct predictions for 73.5% of the drug combinations. The systems for ovary and skin cancer could only correctly predict about 65% of the cases. Unfortunately, the classes of all the drug combinations were incorrectly predicted by the kidney cancer system. In contrast to the specificity scores based on validation data 1, the specificity scores from validation data 2 could be considered more robust as it was evaluated on a far greater number of drug combinations. The predictive systems were measured to perform best when evaluated against the validation data 3 (Fig. 6c). Of the five systems assessed using this dataset, four of them achieved a sensitivity of more than 0.8. The skin cancer predictive system was observed to have a sensitivity of 0.72.

To summarise, it could be commented that the predictive system had varied performance when evaluated against the three external datasets. While comparing these scores, it must be taken into consideration that the external datasets had different sources (validation data 2 and 3) and labelling strategies than the training dataset. The inaccuracies in the assignment of true labels for the drug combinations could be one of the key contributors to the low accuracy of the system. For example, in validation data 1 if a drug combination was synergistic based on only two of the five synergism scores it was labelled as effective unless it had an already reported adverse DDI. On one hand, it might be extrapolated to the fact that the effective labelled drug combinations might not necessarily be effective for all cases of cancer but be more context (e.g., patient-group) specific. On the other hand, the current absence of an adverse DDI report does not completely negate the occurrence of adverse effects in the future. This is also true for validation data 3 which attempted to use a similar strategy to label adverse drug combinations, but none qualified. Ideally, the criteria for assigning true labels in the validation data should match the training data. However, the limited data restricted us. Furthermore, the low specificity in several cases could be linked to the vague information about the kind of adverse effects the adverse drug combinations are associated with. The adverse drug combinations only had reports of an increased risk or severity of adverse effects without the details of the specific type of adverse effect. Due to this, if a drug combination does not sufficiently enrich the selected safety-related features within the predictive system it might not be correctly classified as an adverse drug combination. Thus, highlighting the importance of the inclusion of both features and drug combinations specific to specific adverse effects to train predictive systems for distinguishing effective from adverse drug combinations.

### 3.6 Identification of *de novo* drug combinations

We used our predictive system to identify novel effective and safe drug combinations that could be considered for further experimental validation. The top three selected drug combinations for each cancer type along with their mechanisms of action (MoA) [7] are listed in Table 3. Each of these selected drug combinations received a perfect score of + 1 implying that they were predicted as effective based on all the features included in the predictive system. However, as discussed in the earlier sections, the predictive systems currently do not utilise certain toxicity terms like cardiotoxicity and neurotoxicity for prediction. This can be circumnavigated by directly exploring the efficacy and safety estimates for the drug combinations. For instance, the drug combinations that received a perfect score of + 1 can be prioritised based on the difference in the maximum score of efficacy gene sets and the maximum score of safety gene sets. This ensures that the drug combination has a higher efficacy when compared to the chances of such toxicities. The detailed scores of the top ten drug combinations predicted by our systems with a score of + 1 are given in Appendix A, Appendix A. Within each cancer, a pattern can be observed in the best three-drug combinations selected to be prioritised by our system. In breast cancer, two of the selected combinations were formed by combining an apoptosis stimulant with a hedgehog pathway inhibitor. For lung and ovary cancers, all three selected drug pairs were combinations of an EGFR inhibitor and an HDAC inhibitor. Interestingly, in these two cancers, the same three drug pairs were selected but the order of ranking was different implying that the drug combinations have a slightly different impact on the two cancers. Furthermore, Panobinostat was fixed as the HDAC inhibitor while the EGFR inhibitor varied. Combinations involving growth factor inhibitors were also among the top three drug combinations selected for kidney, prostate and skin cancers. The selected drug combinations could be of particular interest as several of these drug pairs were formed from drugs that were approved only in the last ten years. For example, both arsenic trioxide and glasdegib, whose combination was selected for breast cancer, were approved for marketing in 2018. Similarly, the combination involving asciminib and ivosidenib was prioritised for kidney cancer. The drugs were independently approved for marketing in 2021 and 2018 respectively. Also, the predictive system prioritised combinations of dacomitinib and osimertinib with panobinostat for use in lung and ovary cancers. Panobinostat and osimertinib were approved in 2015 while dacomitinib was approved in 2018. Despite the promising results, caution should be considered as this is a mere prioritisation within a few selected drug pairs. The drug combinations can show a comparatively higher efficacy estimate than the safety estimate resulting in it being predicted as effective. However, when considered independently, they can have sufficiently high safety estimates to show adverse effects. Thus, in the current form of the predictive system, it is recommended that the actual efficacy and safety estimates are also considered along with the final score to make a logical selection of the combinations to be prioritised.

**Table 3:** The list of selected (top three) novel drug combinations to prioritise in the six cancer types. The MoA of the drugs have been retrieved from the Drug Repurposing Hub. Additional details about these drug combinations can be found in Appendix A, Appendix A.

Drug1 DrugBank ID	Drug2 DrugBank ID	Drug1 name	Drug2 name	Drug1 MoA	Drug2 MoA
**Breast Cancer**					
DB06772	DB08901	Cabazitaxel	Ponatinib	Microtubule inhibitor	Bcr-Abl kinase inhibitor; FLT3 inhibitor; PDGFR tyrosine kinase receptor inhibitor
DB01169	DB11978	Arsenic Trioxide	Glasdegib	Apoptosis stimulant	Hedgehog pathway inhibitor
DB01169	DB08828	Arsenic Trioxide	Vismodegib	Apoptosis stimulant	Hedgehog pathway inhibitor; smoothened receptor antagonist
**Kidney Cancer**					
DB12597	DB14568	Asciminib	Ivosidenib	Bcr-Abl kinase inhibitor	Isocitrate dehydrogenase inhibitor
DB08916	DB00694	Afatinib	Daunorubicin, Cytarabine Liposome	EGFR inhibitor	Topoisomerase inhibitor
DB08875	DB00380	Cabozantinib	Dexrazoxane	RET tyrosine kinase inhibitor; VEGFR inhibitor	Chelating agent; topoisomerase inhibitor
**Lung Cancer**					
DB00317	DB06603	Gefitinib	Panobinostat	EGFR inhibitor	HDAC inhibitor
DB09330	DB06603	Osimertinib	Panobinostat	EGFR inhibitor	HDAC inhibitor
DB11963	DB06603	Dacomitinib	Panobinostat	EGFR inhibitor	HDAC inhibitor
**Ovary Cancer**					
DB11963	DB06603	Dacomitinib	Panobinostat	EGFR inhibitor	HDAC inhibitor
DB09330	DB06603	Osimertinib	Panobinostat	EGFR inhibitor	HDAC inhibitor
DB00317	DB06603	Gefitinib	Panobinostat	EGFR inhibitor	HDAC inhibitor
**Prostate Cancer**					
DB06772	DB08875	Cabazitaxel	Cabozantinib	Microtubule inhibitor	RET tyrosine kinase inhibitor; VEGFR inhibitor
DB08875	DB01262	Cabozantinib	Decitabine	RET tyrosine kinase inhibitor; VEGFR inhibitor	DNA methyltransferase inhibitor
DB12267	DB01248	Brigatinib	Docetaxel	ALK tyrosine kinase receptor inhibitor; EGFR inhibitor	Tubulin polymerization inhibitor
**Skin Cancer**					
DB08916	DB00694	Afatinib	Daunorubicin, Cytarabine Liposome	EGFR inhibitor	Topoisomerase inhibitor
DB08916	DB06772	Afatinib	Cabazitaxel	EGFR inhibitor	Microtubule inhibitor
DB15569	DB08881	Sotorasib	Vemurafenib	KRAS inhibitor	RAF inhibitor

Note: The MoA of Sotorasib is currently not reported in the Drug Repurposing Hub. It has been filled based on DrugBank.

### 3.7 Advantages and limitations

A key advantage of the proposed framework is the requirement of minimal information for prediction, i.e., only the drug combination targets are required to predict if it will be effective or adverse. Furthermore, RWR allows the testing of multi-drug combinations using the same framework. Although this work focuses on two-drug combinations, in practicality, it is possible to combine the targets from any number of drugs to predict whether they will be effective or adverse. Another important advantage of the proposed framework is its ability to build a profile for each drug combination based on its relationship to disease-associated genes and adverse effect-associated genes. This simultaneous assessment of efficacy and safety within the same framework allows for a better understanding of the trade-offs between these factors. In addition, as the genes to estimate efficacy and safety are sourced from different databases that focus on different aspects like genetic association, transcriptomic association, etc, the estimates can provide novel insight into the mechanisms underlying effective and safe drug combinations. Despite these advantages, our framework is not devoid of limitations. The accuracy of true labels in the training data is critical to developing a robust predictive system. For example, the current setup is penalised because effective drug combinations in the training data are labelled based on the absence of reported adverse DDI, which may not reflect their true status. Additionally, the drug combinations that qualified to be adverse were mostly associated with an increased risk or severity of adverse effects without specifying the actual effect. This restricted the correct identification of drug combinations with very specific side effects. Furthermore, it was observed that the predictive system performs well within the scope of chemical subgroup pairing included in the training data. To extend the scope, it might be necessary to expand the dataset with drug combinations from other chemical subgroups. Finally, though we developed separate predictive systems for the different cancer types, it does not account for the differences in patient subgroups that may lead to differences in drug combination effect. In future versions of our framework, we aim to improve particularly on this aspect to develop a predictive system for prioritisation of cancer sub-type specific drug combinations.

## 4 Conclusions

In this work, we developed a computational tool using a network medicine framework to identify and prioritise effective and safe anti-cancer drug combinations. Our proof-of-concept evaluates the mechanistic efficacy and safety of drug pairs through a combination of RWR and FGSEA approaches. We integrated a multitude of publicly available interaction/association data to develop a case study on six cancer types demonstrating how the efficacy and safety estimates could be utilised to build predictive systems for identifying effective and safe anti-cancer drug combinations – moving away from the traditional synergistic and antagonistic anti-cancer drug combination identification. Through rigorous internal and external validation, we show that while our approach holds potential, the actual success relies on how accurate the true labels for the drug combinations used for training are. We believe our predictive systems could have even better accuracy in predicting effective and safe anti-cancer drug combinations by further improving the labels of the training set drug combinations and incorporating more robust gene sets of efficacy and safety.

## Abbreviations

ATC: Anatomical Therapeutic Chemical.

CDCDB: Continuous Drug Combination Database.

CTD: Comparative Toxicogenomics Database.

DDI: Drug-Drug Interactions.

EMA: European Medicines Agency.

FDA: Food and Drug Administration.

FGSEA: Fast Gene Set Enrichment Analysis.

HSA: Highest Single Agent.

NES: Normalised Enrichment Scores.

PPI: Protein-protein interaction.

RWR: Random Walk with Restart.

ZIP: Zero Interaction Potency.

## Authorship responsibility

All authors have reviewed and approved the content of the submitted manuscript. All authors have agreed on the authorship, the order of authors, the naming of the corresponding author for all correspondence with the publisher, and the publication of the article.The manuscript presents original work that has not been previously published in a similar form and is not currently under consideration by another journal.

## Funding sources

This work was supported by the 10.13039/501100004012Jane and Aatos Erkko Foundation (210026) and the 10.13039/501100006306Sigrid Jusélius Foundation.

## CRediT authorship contribution statement

**Vittorio Fortino:** Writing – review & editing, Writing – original draft, Supervision, Resources, Project administration, Methodology, Investigation, Funding acquisition, Formal analysis, Conceptualization. **Arindam Ghosh:** Writing – review & editing, Writing – original draft, Software, Methodology, Formal analysis, Data curation, Conceptualization.

## Declaration of Competing Interest

The authors declare that there is no conflict of interest.

## Data Availability

Publicly accessible data were used in the study. The R scripts for analyses of the data are available on GitHub (https://github.com/UEFBiomedicalInformaticsLab/NEDEA).

## Footnotes

Appendix A Supplementary data associated with this article can be found in the online version at doi:10.1016/j.csbj.2024.12.003.
