# Supplementary material for: Network-based estimation of therapeutic efficacy and adverse reaction potential for prioritisation of anti-cancer drug combinations
Source: Comput Struct Biotechnol J. 2024 Dec 7;27:65–77. doi: 10.1016/j.csbj.2024.12.003 (PMC12604529; doi:10.1016/j.csbj.2024.12.003)

## Slide 1
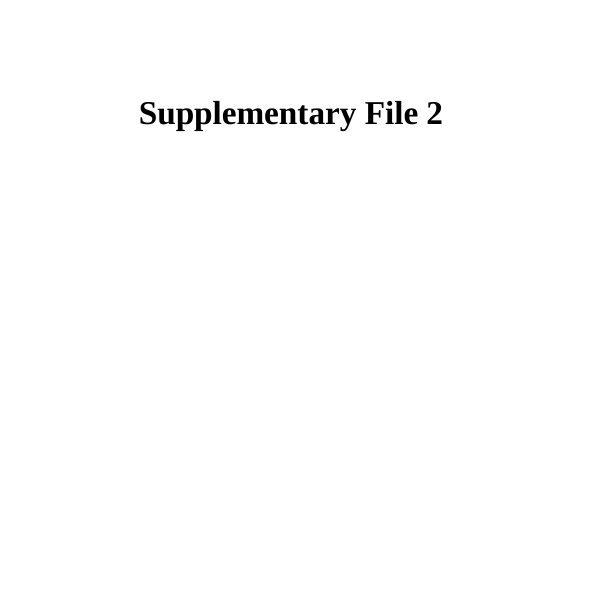

Supplementary File 2

## Slide 2
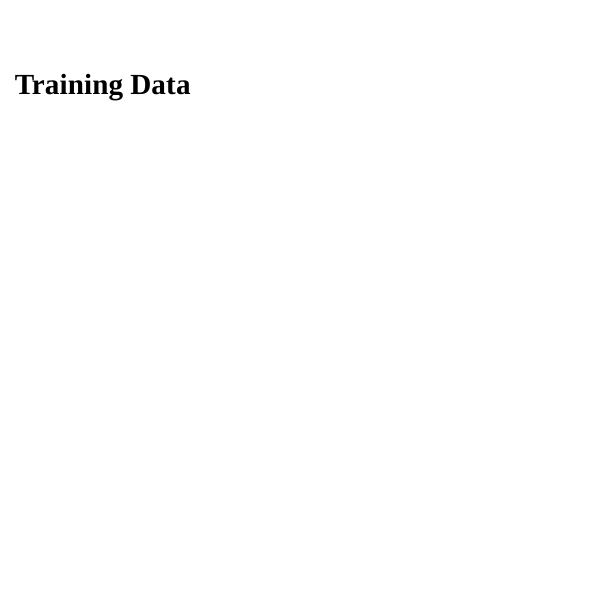

Training Data

## Slide 3
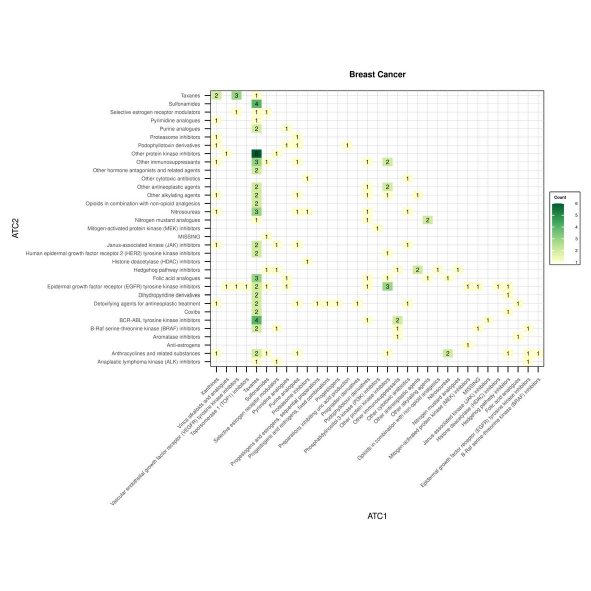

## Slide 4
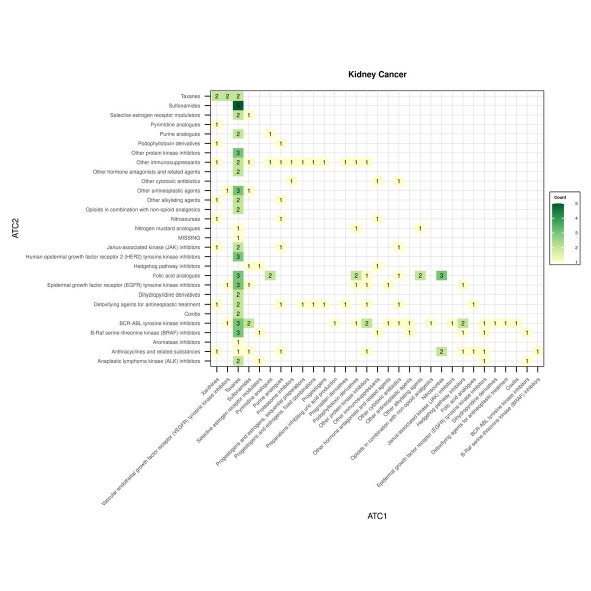

## Slide 5
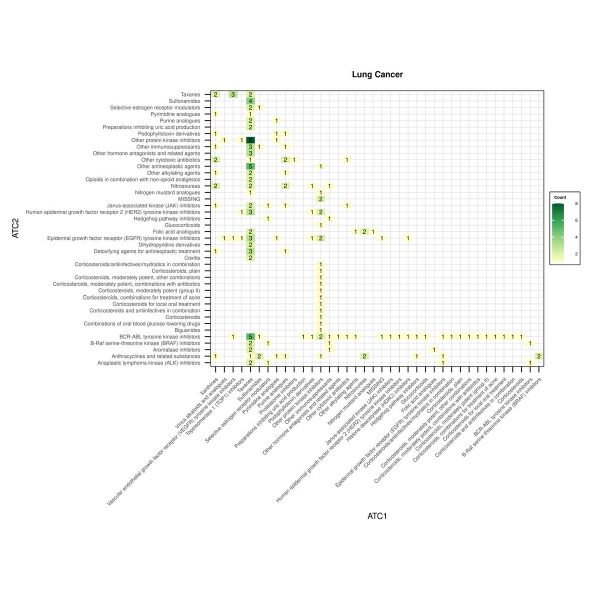

## Slide 6
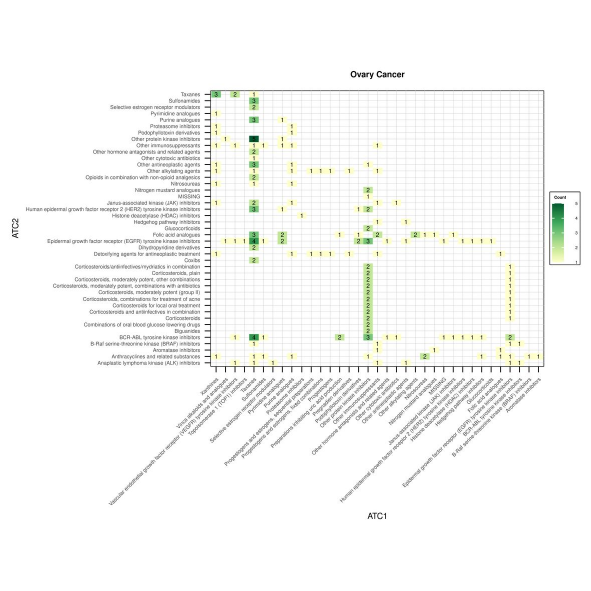

## Slide 7
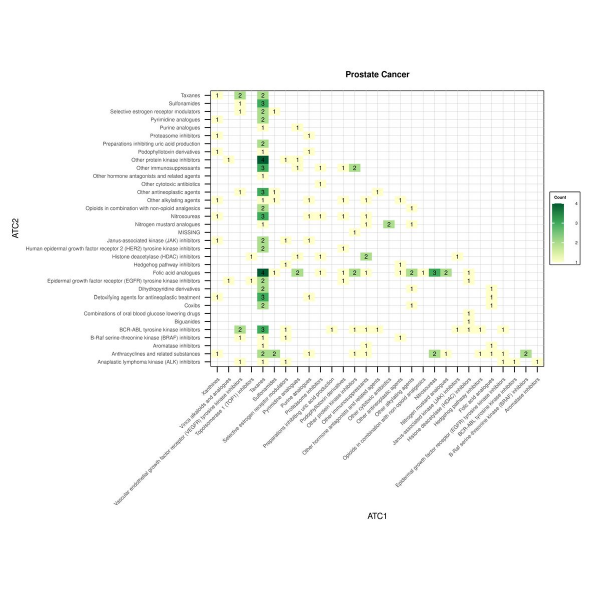

## Slide 8
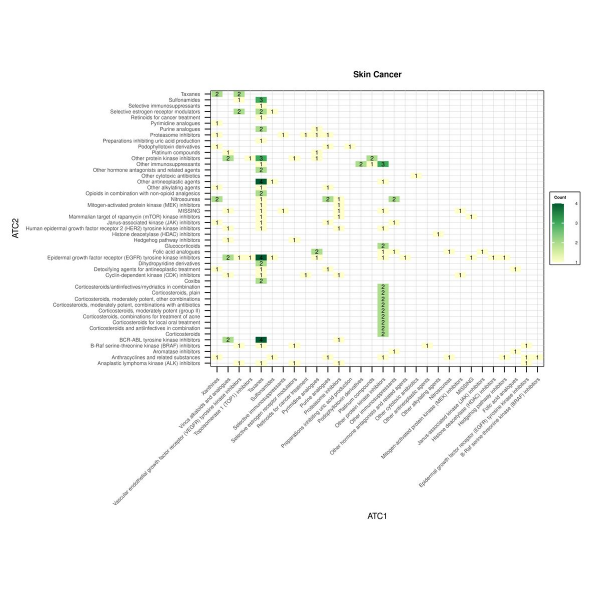

## Slide 9
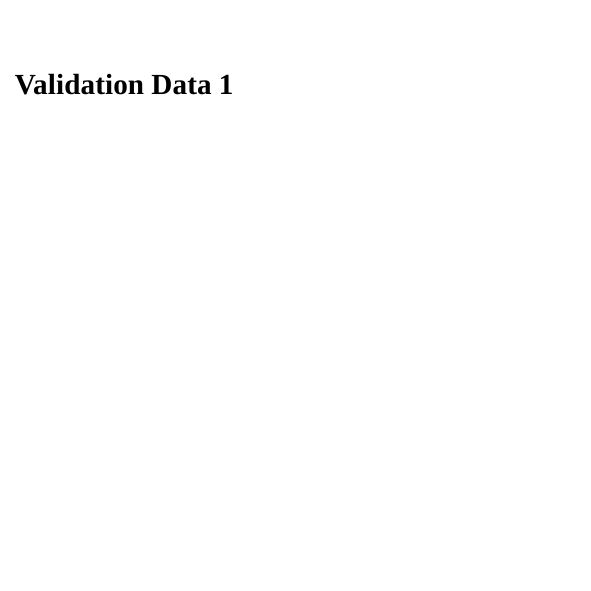

Validation Data 1

## Slide 10
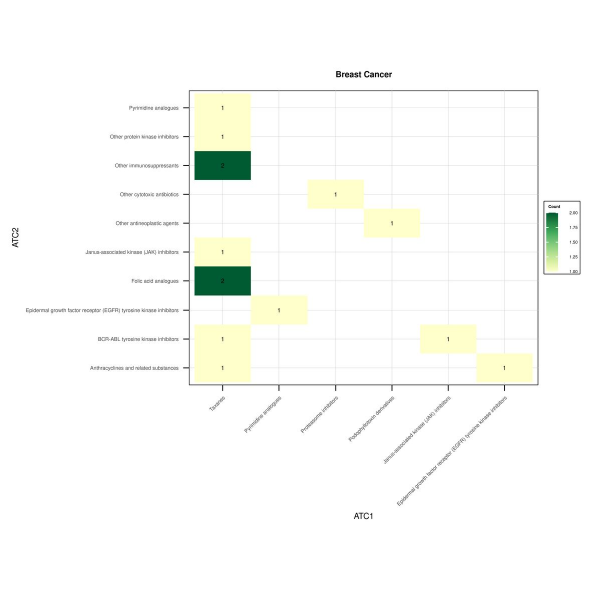

## Slide 11
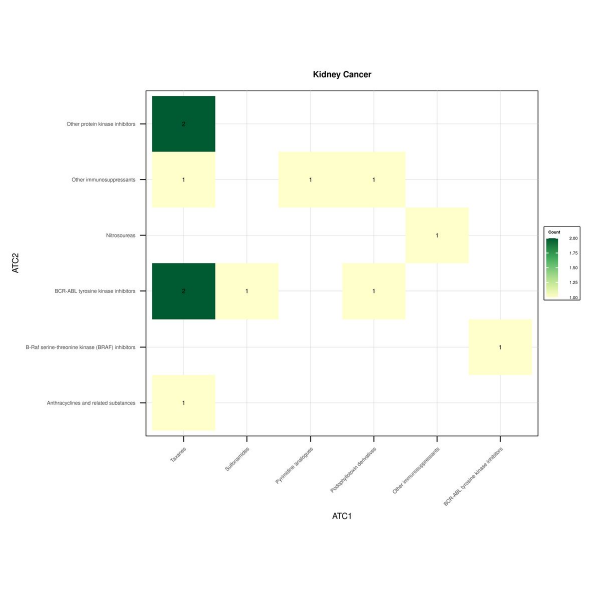

## Slide 12
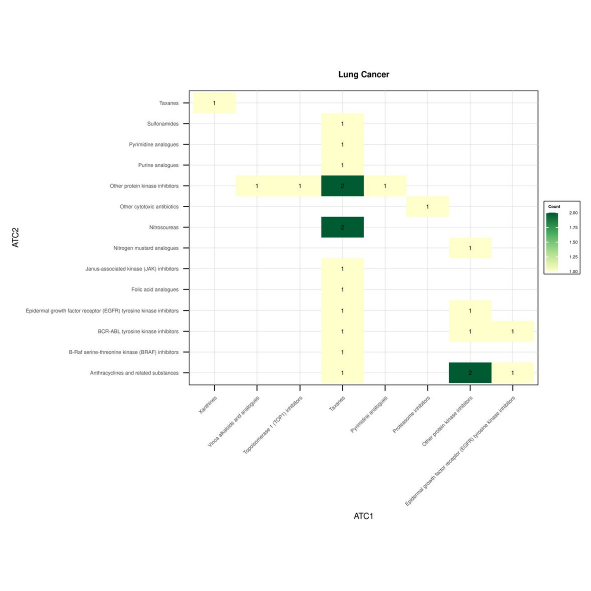

## Slide 13
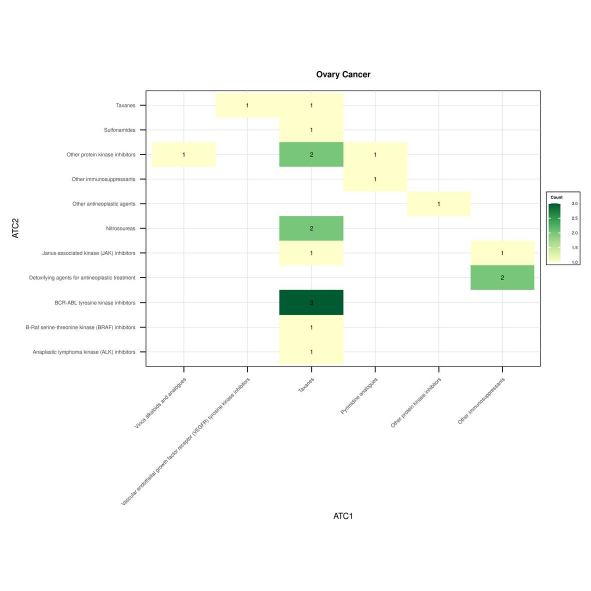

## Slide 14
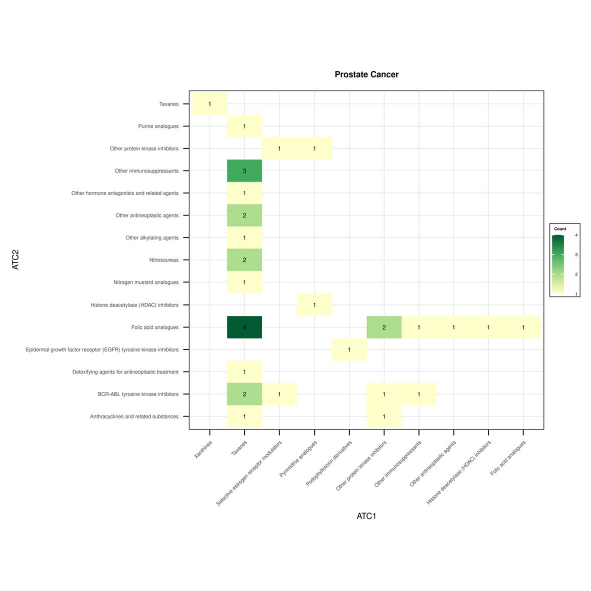

## Slide 15
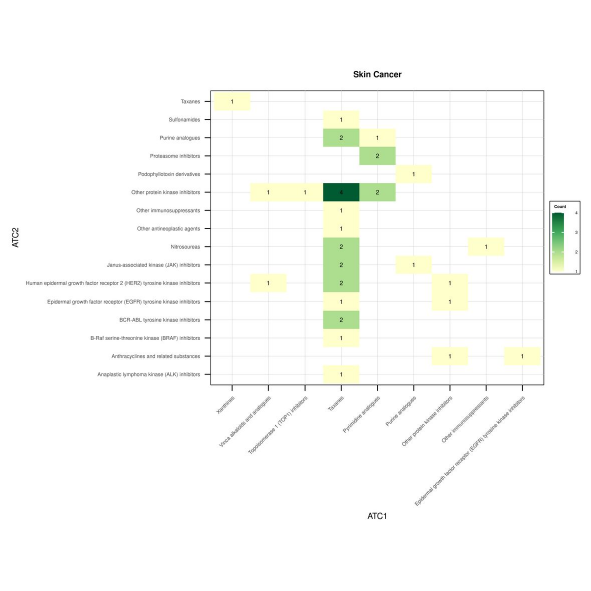

## Slide 16
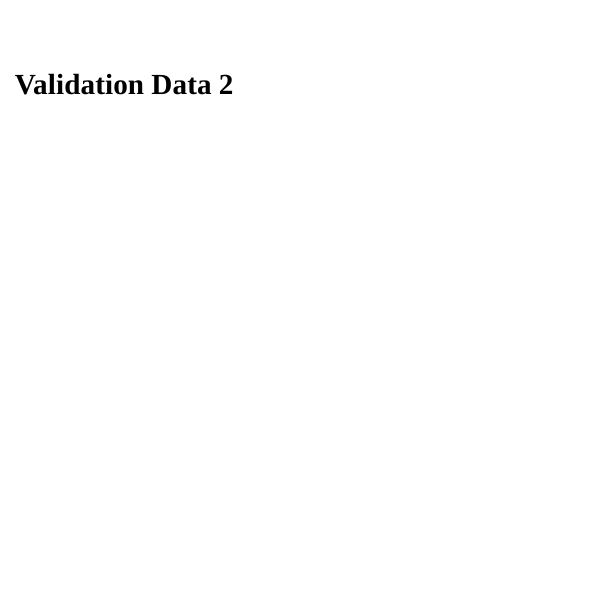

Validation Data 2

## Slide 17
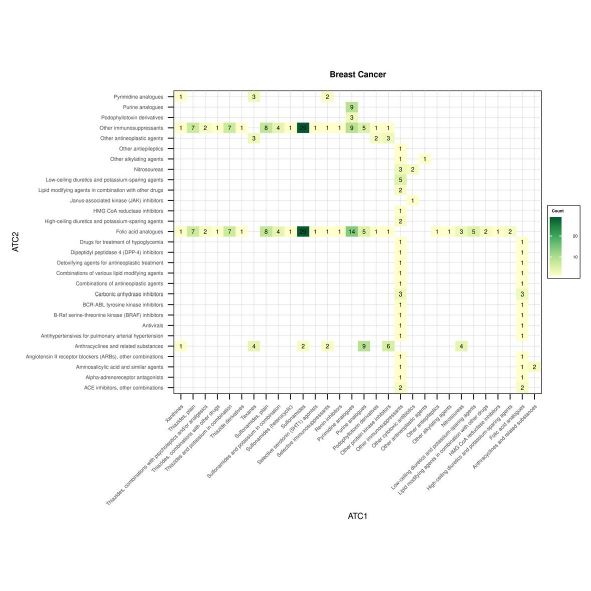

## Slide 18
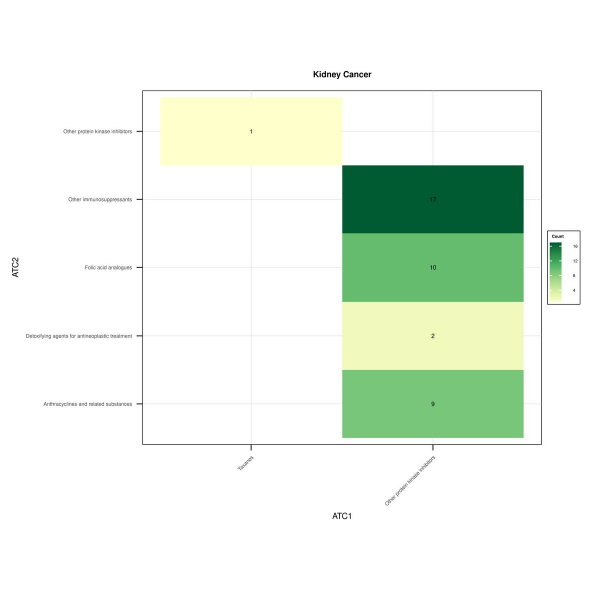

## Slide 19
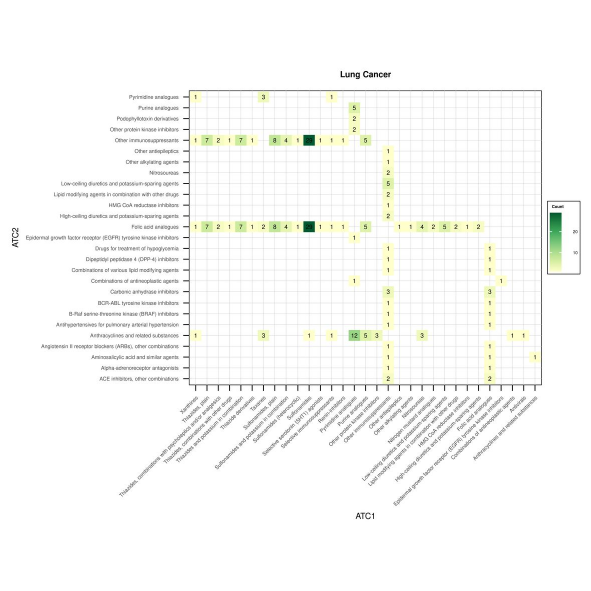

## Slide 20
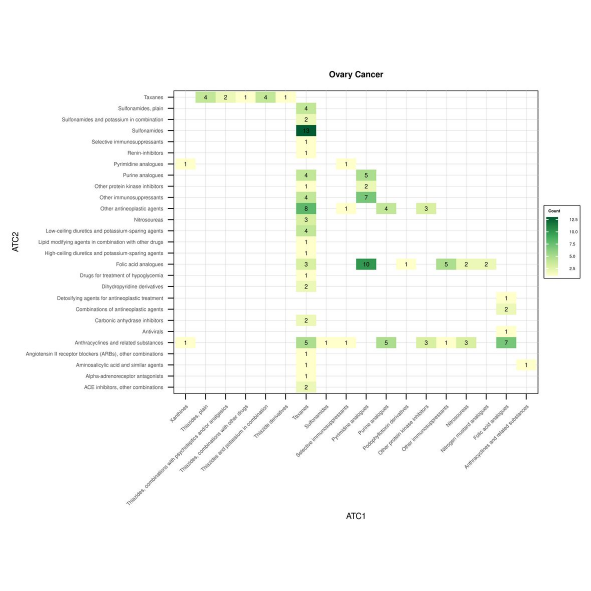

## Slide 21
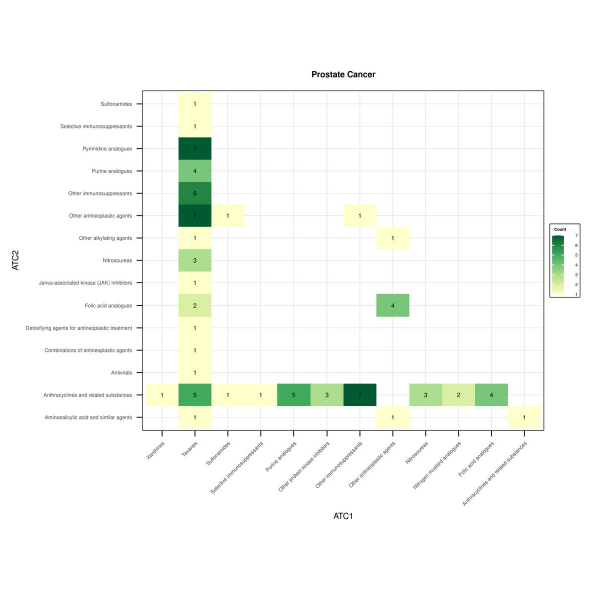

## Slide 22
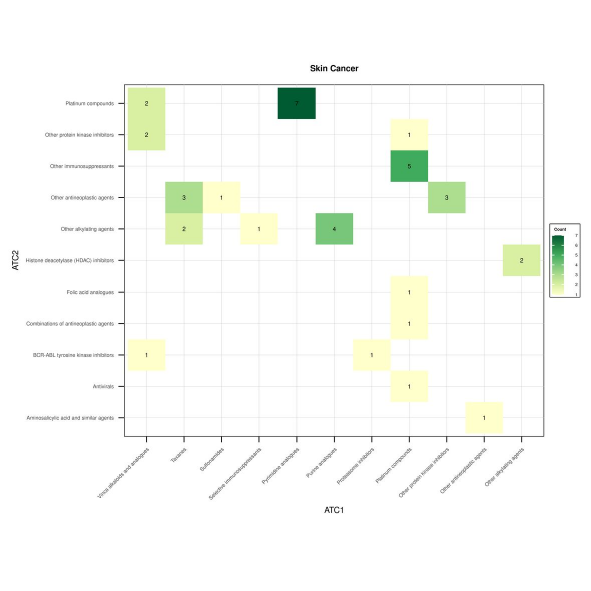

## Slide 23
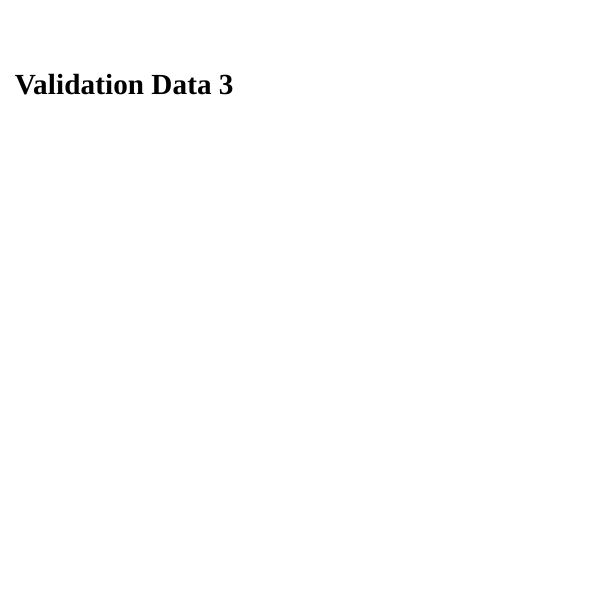

Validation Data 3

## Slide 24
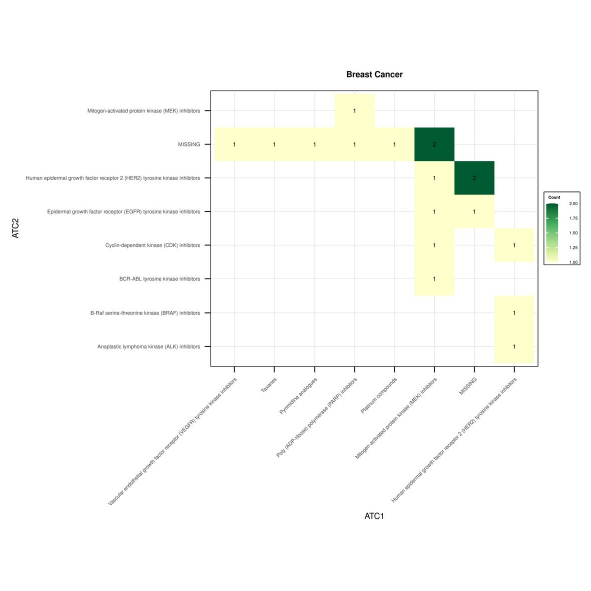

## Slide 25
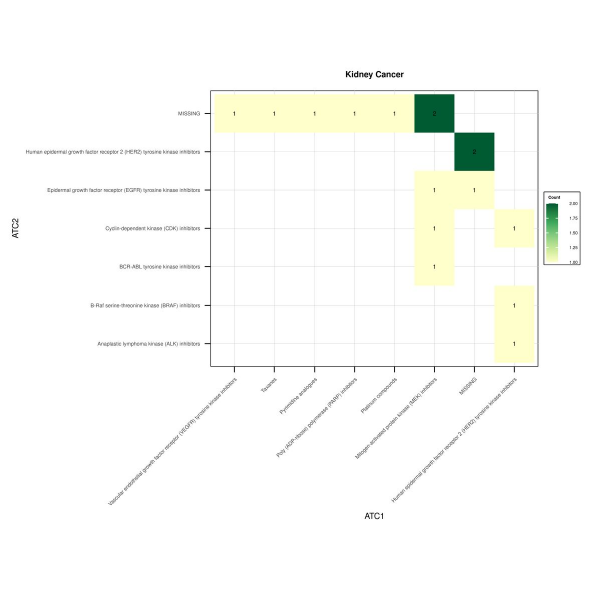

## Slide 26
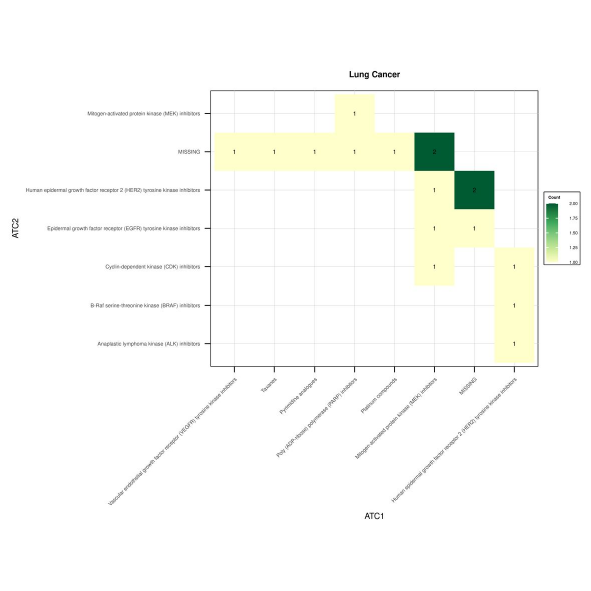

## Slide 27
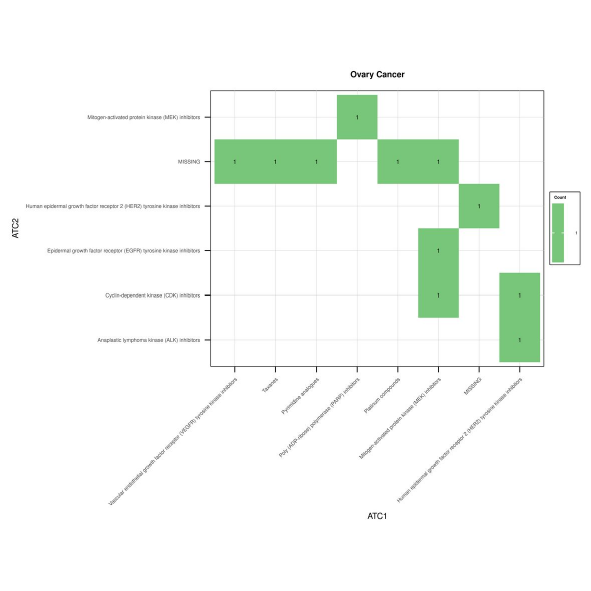

## Slide 28
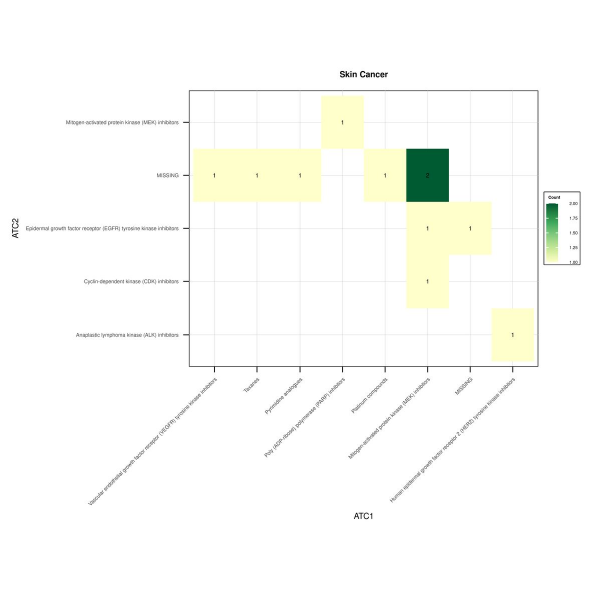

## Slide 29
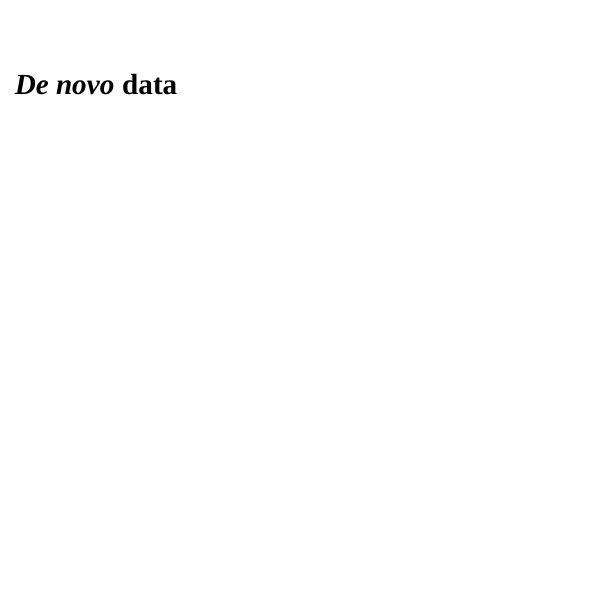

De novo data

## Slide 30
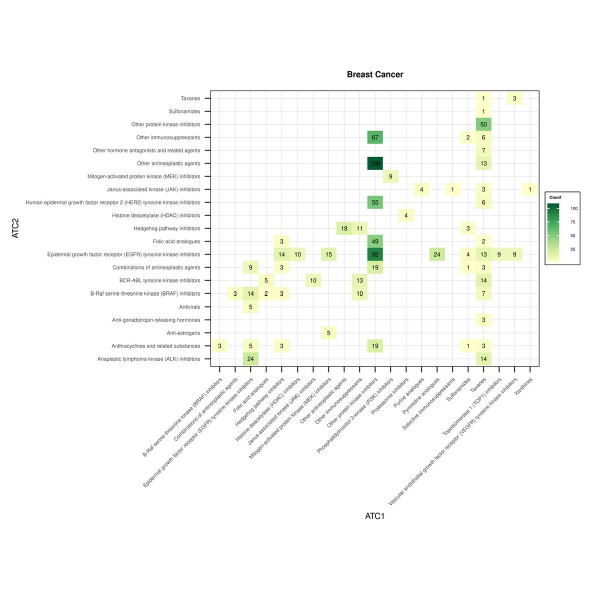

## Slide 31
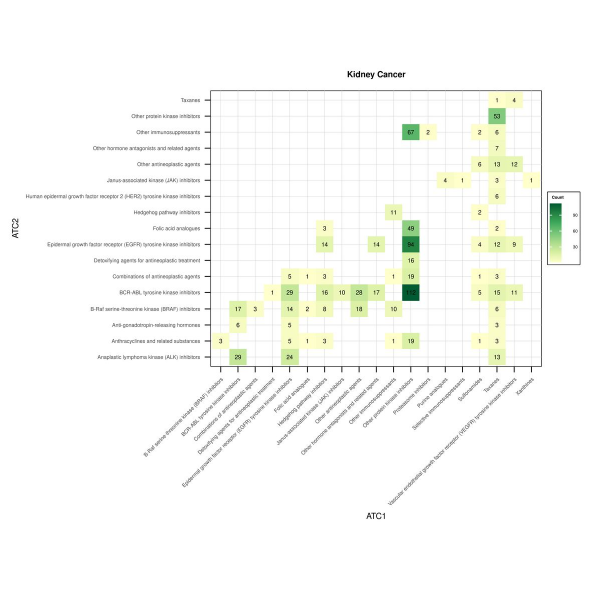

## Slide 32
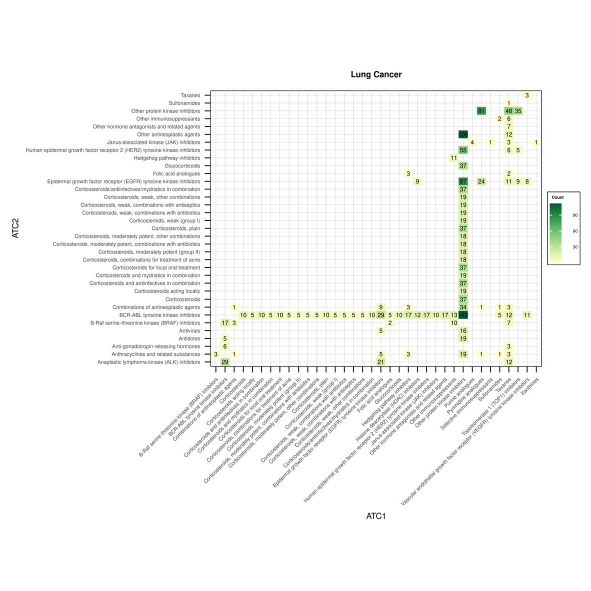

## Slide 33
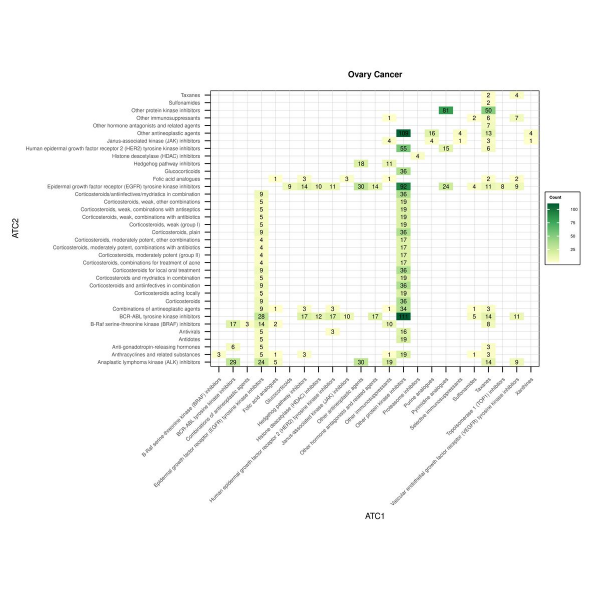

## Slide 34
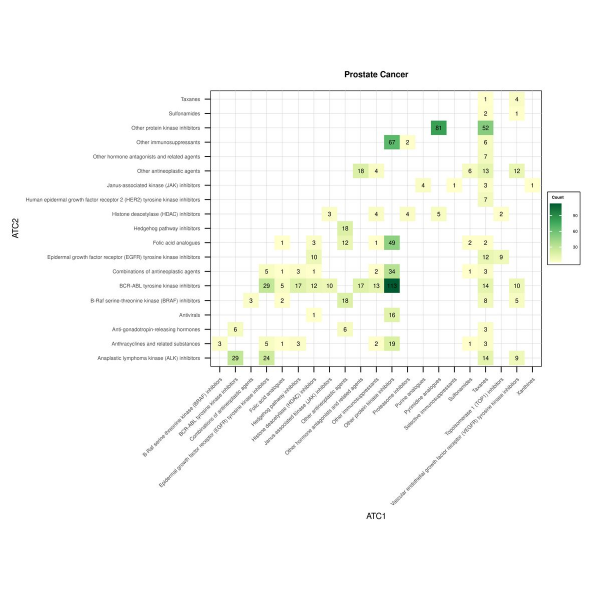

## Slide 35
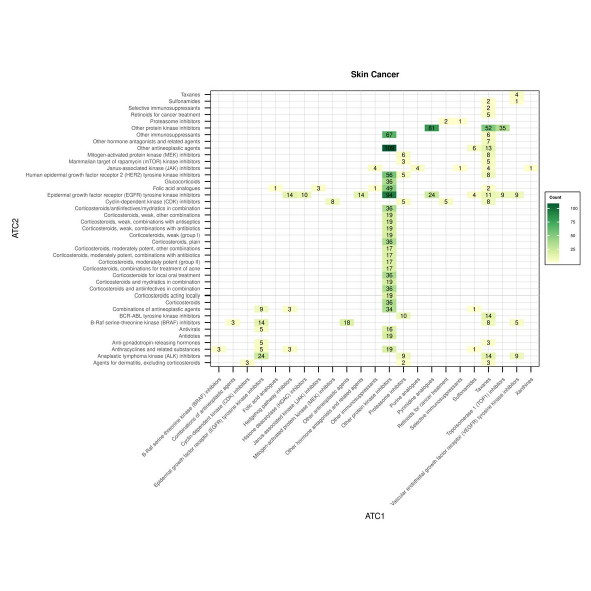

Supplement: Supplementary file 1 — Supplementary material [file mmc1.zip › Supplementary_Files/Supplementary_File_2_30Nov2024.pptx]
