# Supplementary figures and images for: Network-based estimation of therapeutic efficacy and adverse reaction potential for prioritisation of anti-cancer drug combinations
Source: Comput Struct Biotechnol J. 2024 Dec 7;27:65–77. doi: 10.1016/j.csbj.2024.12.003 (PMC12604529; doi:10.1016/j.csbj.2024.12.003)

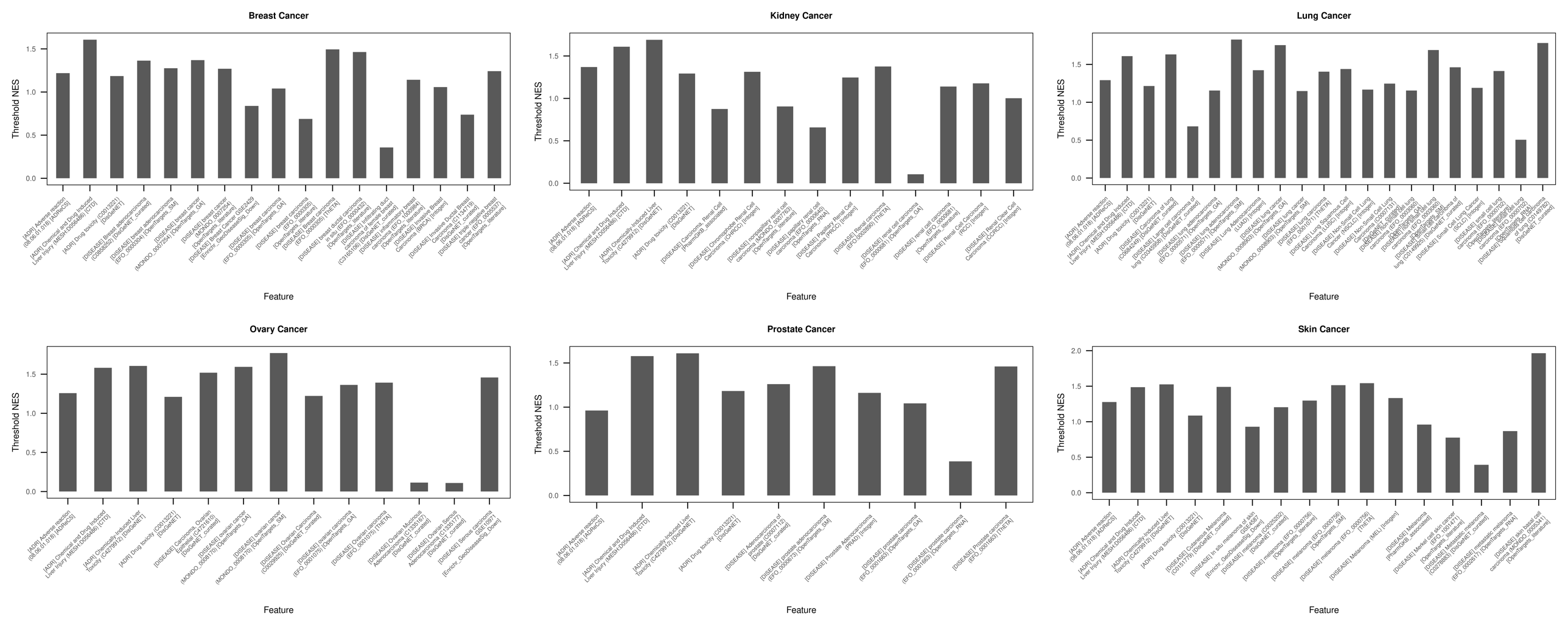

Supplement: Supplementary file 2 — Supplementary material [file mmc2.zip › Supplementary_Figures/Supplementary_Figure_6_30Nov2024.tiff]

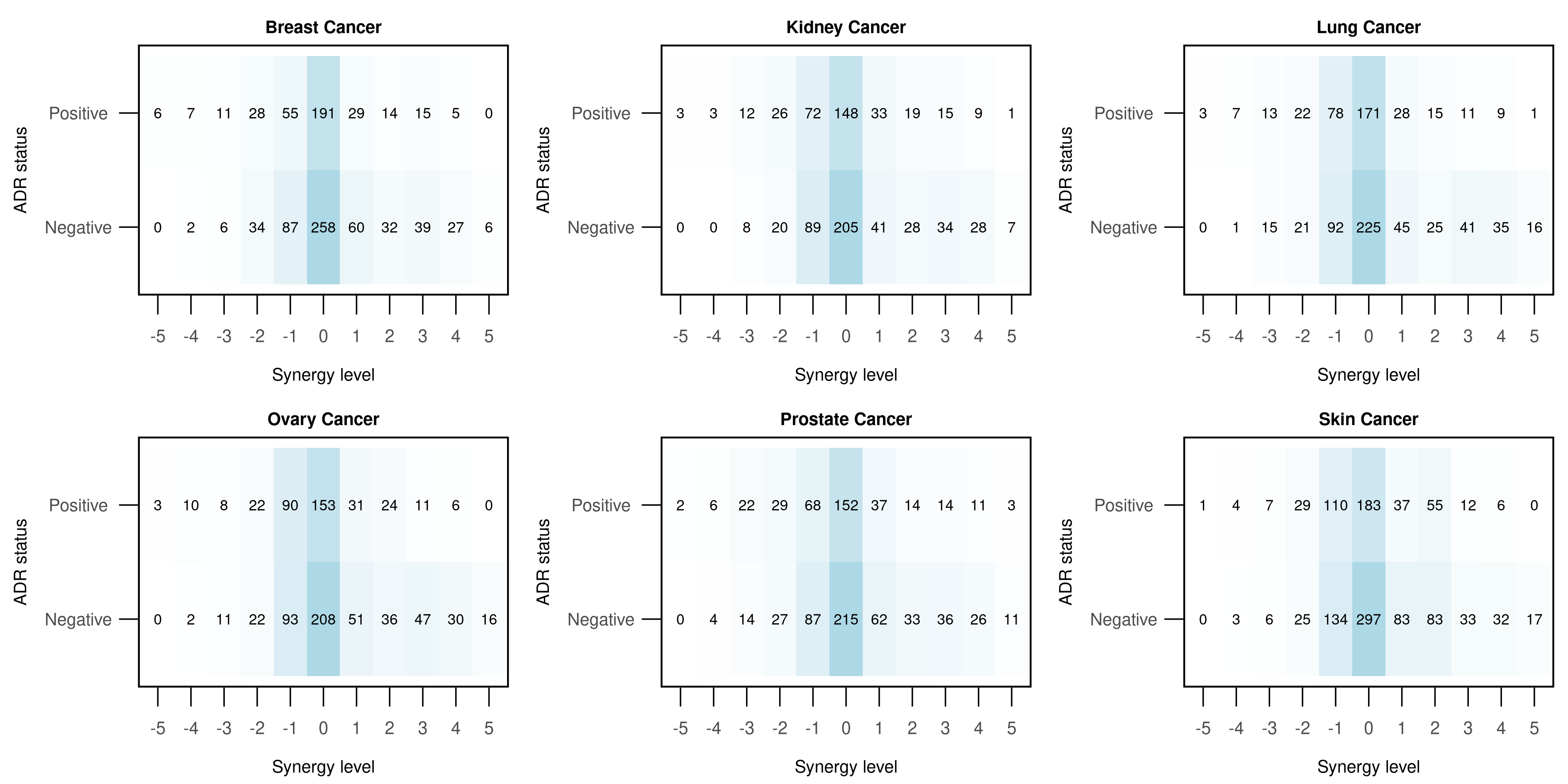

Supplement: Supplementary file 2 — Supplementary material [file mmc2.zip › Supplementary_Figures/Supplementary_Figure_1_30Nov2024.tiff]

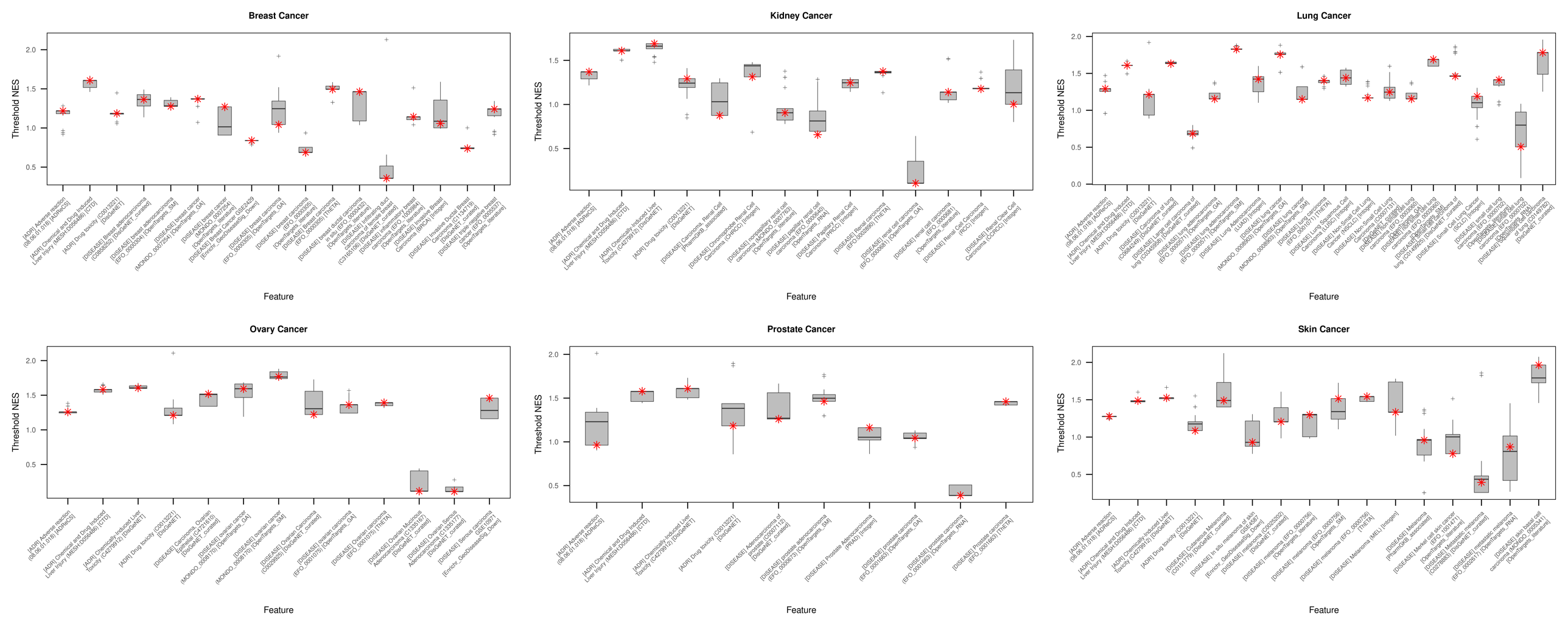

Supplement: Supplementary file 2 — Supplementary material [file mmc2.zip › Supplementary_Figures/Supplementary_Figure_7_30Nov2024.tiff]

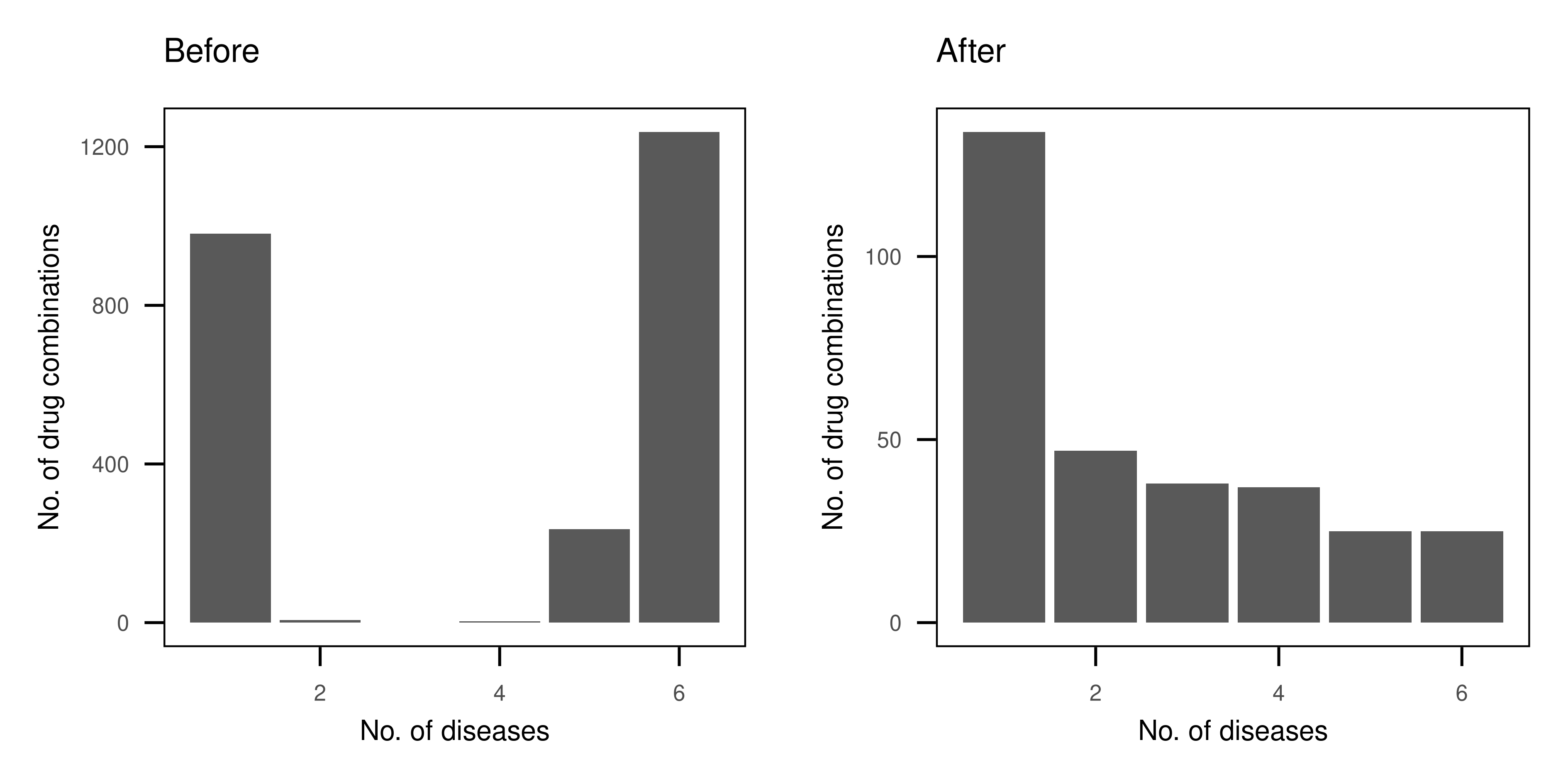

Supplement: Supplementary file 2 — Supplementary material [file mmc2.zip › Supplementary_Figures/Supplementary_Figure_2_30Nov2024.tiff]

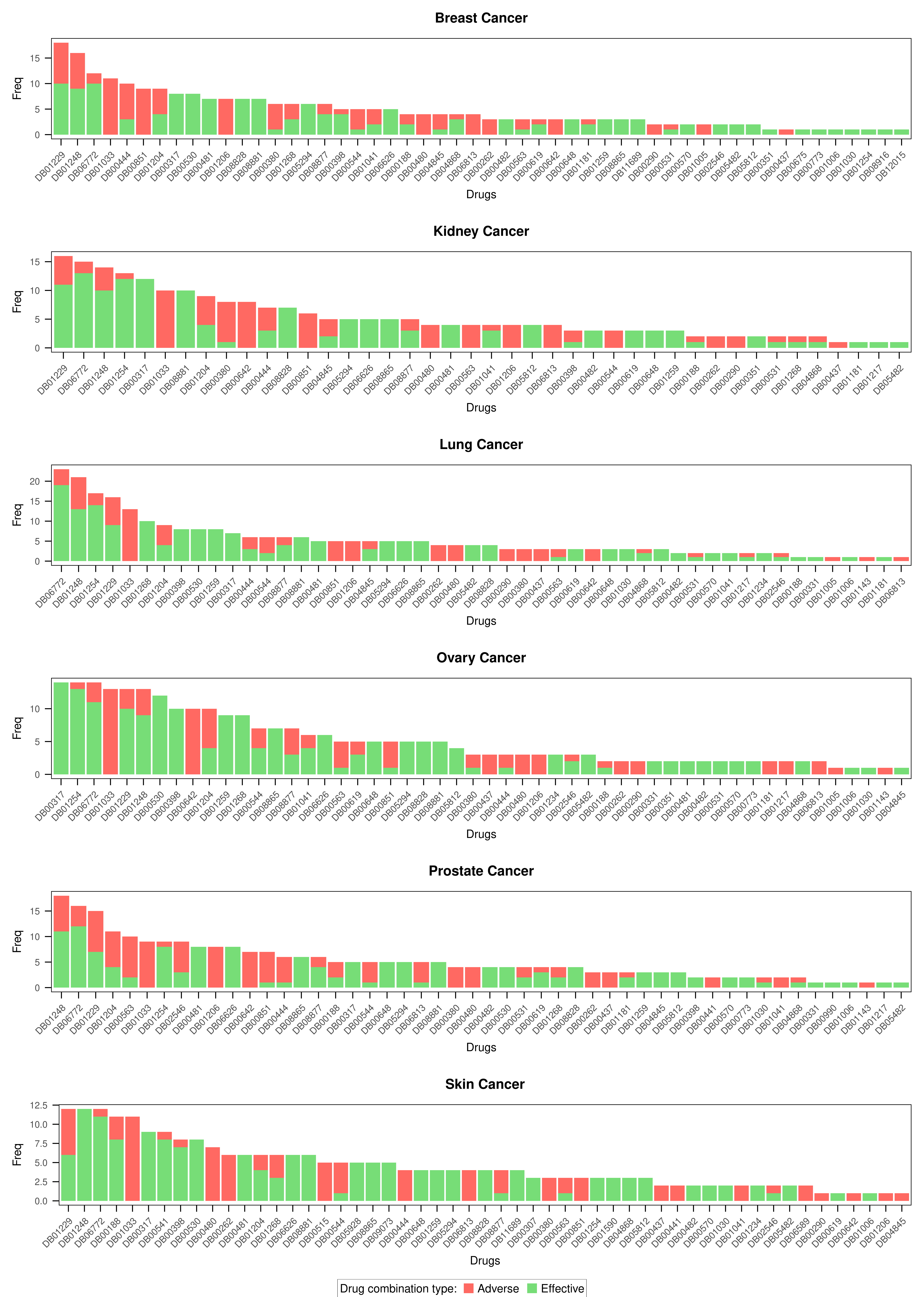

Supplement: Supplementary file 2 — Supplementary material [file mmc2.zip › Supplementary_Figures/Supplementary_Figure_3_30Nov2024.tiff]
