# Supplementary material for: Network-based estimation of therapeutic efficacy and adverse reaction potential for prioritisation of anti-cancer drug combinations
Source: Comput Struct Biotechnol J. 2024 Dec 7;27:65–77. doi: 10.1016/j.csbj.2024.12.003 (PMC12604529; doi:10.1016/j.csbj.2024.12.003)
Supplement: Supplementary file 1 — Supplementary material [file mmc1.zip › Supplementary_Files/Supplementary_File_description.doc]

**Supplementary Files**

**Supplementary File 1:** Composition of the efficacy and safety gene set libraries. For the six cancers, we have separate gene sets for each cancer. The safety gene set is common for all cancers. The table summarises the number of genes in each gene set compiled from the databases and those in the PPI network.

**Supplementary File 2:** Paired chemical sub-groups in the different datasets. The heatmaps show the number of drug combinations per paired chemical sub-group. If a drug belongs to more than one chemical sub-group (as in ATC classification level 4) it is represented in all the relevant groups. Only the paired chemical sub-groups present in the training dataset are used to select drug combinations for validation (except dataset 3) and de novo datasets.

**Supplementary File 3:** List of features showing statistically significant differences in the distribution of efficacy/safety estimates between the groups of drug combinations.

**Supplementary File 4:** List of features showing statistically significant differences in the distribution of separation distances (proximity) between the groups of drug combinations.

**Supplementary File 5:** List of top ten (if any) novel drug combinations for each cancer type selected by our predictive system and prioritised by the differences of maxs approach. The list includes details about the individual drugs for the combination like drug name, approval status and ATC labels. The columns ‘which_max_efficacy_NES’ and ‘which_max_safety_NES’ mention the efficacy and safety gene set with the highest estimate for the drug combination. These columns can be used to infer the specific cancer type the drug combination may be effective against or the adverse effect it may cause. The actual estimates of the features are given in columns ‘max_efficacy_NES’ and ‘max_safety_NES’. The ‘diff_of_maxs’ columns give the difference in the maximum efficacy and safety estimate for the drug combinations and have been used for prioritisation.
